# Boundary-aware and uncertainty-guided deep learning for multimodal magnetic resonance imaging segmentation of stroke lesions

**DOI:** 10.3389/fneur.2026.1872532

**Published:** 2026-08-04

**Authors:** Zhongxiang Xing, Yuding Luo, Yupeng Niu, Yilin Yang, Zhenzhen Lin, Han Qin, Zhiyin Chen, Jian Wang, Jiong Mu

**Affiliations:** 1 College of Information Engineering, Sichuan Agricultural University, Ya’an, China; 2 Department of Neurology, Ya’an People’s Hospital, Sichuan Province, Ya’an, China; 3 Tianjin Key Laboratory of Radiation Medicine and Molecular Nuclear Medicine, Institute of Radiation Medicine, Chinese Academy of Medical Sciences and Peking Union Medical College, Tianjin, China; 4 NewIdea Yunshu Technology Co., Ltd., Chengdu, China; 5 Tsinghua University, Beijing, China; 6 Department of Neurology, The Affiliated Hospital, Southwest Medical University, Luzhou, China

**Keywords:** acute ischemic stroke, cerebrovascular disease, deep learning, graph attention, lesion segmentation, multimodal magnetic resonance imaging, uncertainty estimation

## Abstract

**Introduction:**

Acute ischemic stroke is a major cerebrovascular disease, and accurate lesion segmentation is important for quantitative image analysis and subsequent clinical assessment. Multimodal magnetic resonance imaging, including diffusion-weighted imaging, apparent diffusion coefficient maps, and fluid-attenuated inversion recovery, provides complementary information for lesion delineation. However, lesion appearance may vary across sequences, and blurred boundaries or small scattered lesions can make multimodal feature fusion unstable.

**Methods:**

We developed MBGS-UNet, a multi-branch deep learning model for acute ischemic stroke lesion segmentation. Separate encoders were used to preserve sequence-specific information. A boundary-aware graph attention module was introduced at deeper feature levels to regulate cross-modal interaction in ambiguous regions, and an uncertainty-gated fusion module was used to balance graph-enhanced features with encoder features at the voxel level. Uncertainty-weighted boundary supervision and consistency learning were further incorporated to emphasize difficult regions during training. The model was evaluated on the ISLES-2022 dataset using an independent test set and label-free sliding-window inference.

**Results:**

MBGS-UNet achieved a Dice score of 0.7208 on the independent test set, numerically higher than M2FNet (0.7068) and UMamba (0.7016) under the same evaluation protocol. However, case-wise paired statistical analysis did not provide sufficient evidence to support clear superiority over these strong baselines. Visual analysis showed that high-uncertainty responses were mainly concentrated around lesion boundaries and other error-prone regions, indicating a spatial relationship between predictive uncertainty and local segmentation difficulty.

**Discussion:**

MBGS-UNet achieved competitive segmentation performance while providing interpretable uncertainty visualization. The uncertainty maps may offer useful cues for identifying ambiguous lesion boundaries and other regions that require closer review. Nevertheless, the observed numerical improvement should not be interpreted as conclusive evidence of superiority or clinical validation. Further evaluation on larger, more diverse, and external cohorts is required to establish broader robustness and clinical applicability.

## 1 Introduction

Accurate segmentation of acute ischemic stroke lesions is important for quantitative stroke imaging analysis and clinical assessment. Multimodal MRI, including DWI, ADC, and FLAIR, provides complementary information through different imaging mechanisms (1), making multimodal fusion an important strategy for improving segmentation accuracy (2). However, lesion visibility can vary across sequences. Lesion boundaries are often blurred, irregular, and low in contrast, and small scattered lesions may be overlooked. These factors make simple concatenation or fixed-weight fusion less reliable for adapting to regional differences in segmentation difficulty. In cerebrovascular imaging, automatic lesion segmentation can support quantitative assessment by reducing variability in lesion delineation, but this task remains challenging because lesion visibility often differs across MRI sequences, especially near blurred infarct boundaries.

Existing multimodal segmentation methods typically employ strategies such as early fusion, late fusion, or multi-branch fusion, yet two issues remain prevalent: On one hand, cross-modal semantic conflicts are most pronounced in boundary regions. Direct strong interactions may propagate noise, while excessive suppression of interactions leads to over-smoothed edges and loss of fine details. On the other hand, many approaches lack explicit modeling of “feature credibility,” making it difficult to determine where to rely more on complementary information versus preserving original details. Uncertainty estimation provides a natural signal for characterizing prediction reliability (3), but it is currently used more for post-inference quality control and less systematically integrated into fusion pipelines and training objectives.

From the perspective of artificial intelligence-assisted cerebrovascular image analysis, this study focuses on three issues that commonly affect acute ischemic stroke lesion segmentation: cross-modal inconsistency, boundary ambiguity, and uncertain local predictions. Rather than framing the method as a definitive state-of-the-art accuracy model, we aimed to develop an uncertainty-aware segmentation framework that can maintain competitive accuracy while providing interpretable information about difficult regions. To this end, we developed MBGS-UNet, a multi-branch model that incorporates boundary-aware graph interaction and uncertainty-guided fusion. Experiments on the ISLES-2022 dataset were conducted under a unified evaluation setting (4), and uncertainty visualizations were used to examine whether high-uncertainty regions corresponded to lesion boundaries and local prediction errors.

In the segmentation task for acute ischemic stroke, a single modality struggles to simultaneously characterize both the infarct core and the ischemic penumbra (5). Consequently, fusing multimodal MRI (such as DWI, ADC, and FLAIR) has become the mainstream strategy. Based on the timing of fusion, this approach can be categorized into three types: early fusion, late fusion, and multi-branch fusion.

### 1.1 Early fusion

Directly concatenating different modalities along the channel dimension before feeding them into a single stream network, as in DeepMedic (6). This approach is simple to implement but implicitly assumes that all modalities follow similar spatial and intensity distributions. It overlooks imaging mechanism differences—such as high signal in DWI versus low signal in ADC—and is prone to generating cross-modal feature interference in deep semantic spaces.

### 1.2 Late fusion

Represented by the work of Havaei et al. (7), this strategy involves training networks independently for each modality, then performing weighted averaging or voting fusion at the decision layer (e.g., before the Softmax output). While this approach avoids feature-level interference, it also severs the connections between modalities at the low-level texture and mid-level semantic levels, making it difficult to model complex nonlinear complementary relationships.

### 1.3 Multi-branch networks

The multi-encoder architecture extracts features from each modality through independent encoders, with interactions occurring at intermediate layers via skip connections or attention modules. Compared to the previous two approaches, this method better preserves modality-specific information. However, existing work often employs simple element-wise summation or concatenation, lacking dynamic evaluation of feature reliability. To address this limitation, this paper introduces uncertainty-driven modal gating and graph attention within the multi-encoder framework, enabling adaptive filtering and fusion of multimodal features (2).

Traditional convolutional neural networks (CNNs) are constrained by their local receptive fields, leading to inadequate modeling of long-range dependencies within complex brain structures. Graph neural networks (GNNs) better capture global context by treating voxels or feature units as graph nodes (8) and propagating information in non-Euclidean space.

Graph Attention Networks (GAT) further introduce self-attention mechanisms (9), adaptively assigning aggregation weights based on node feature similarity. In multimodal scenarios, Multimodal Interaction Graph Attention (MIGGAT) treats different modalities as graph nodes, leveraging cross-modal attention to model modal correlations and complementarities.

However, existing GAT/MIGGAT models generally rely on the “smoothness assumption,” tending to suppress interactions between nodes with significant differences to reduce noise. In stroke lesion segmentation, lesion boundaries often exhibit strong intermodal conflicts (e.g., lesions are visible on DWI but not yet clear on FLAIR). Simply suppressing such differences can lead to excessive smoothing of critical edge information (10, 11). To address this, we propose the Boundary-Aware Modality Interaction Graph Attention (Boundary-Aware MIGGAT). This module suppresses high uncertainty propagation in non-boundary regions while leveraging intermodal uncertainty differences in boundary regions to enhance boundary features.

Deep neural network predictions often exhibit excessive “confidence” (3), lacking characterization of their own reliability. Therefore, uncertainty estimation holds significant importance in clinical applications.

Bayesian Approximation and MC Dropout: Bayesian neural networks (BNNs) provide theoretically rigorous uncertainty estimation but incur substantial computational overhead. Monte Carlo (MC) Dropout approximates Bayesian inference by repeatedly sampling and averaging results during inference. While effective, it significantly increases inference time, hindering real-time applications (12).

Entropy-Based Deterministic Quantization: Shannon entropy based on the predicted probability distribution serves as a computationally efficient uncertainty metric. It requires no structural changes to the network or multiple forward passes, making it suitable for integration into existing segmentation frameworks (13).

Applications in segmentation: Current work predominantly employs uncertainty for post-processing quality control or active learning sample selection, with limited direct involvement in feature extraction and fusion. Some studies utilize uncertainty to weight loss functions, yet systematic application in multimodal fusion and consistency learning remains scarce (14). Unlike previous studies that mainly use uncertainty after prediction, our method brings uncertainty into both feature fusion and model training. In the proposed framework, uncertainty is not treated only as an auxiliary map for later inspection. Instead, it is involved in graph-based interaction and teacher-student consistency learning, allowing the network to pay more attention to difficult samples and regions where the modalities disagree. In this way, uncertainty helps the model improve segmentation while also making unreliable regions easier to identify and interpret.

## 2 Materials and methods

### 2.1 Methodology

Segmenting acute ischemic stroke lesions from multimodal magnetic resonance imaging remains difficult because different imaging sequences may show the same lesion with inconsistent contrast, and lesion boundaries are often indistinct. We designed MBGS-UNet as a multi-branch deep learning model for acute ischemic stroke lesion segmentation in cerebrovascular magnetic resonance imaging. Each sequence is first encoded separately, and cross-modal interaction is then introduced at deeper layers, where semantic conflicts are more likely to occur. This design preserves sequence-specific information while allowing the model to handle ambiguous regions through boundary-aware fusion.

#### 2.1.1 Overall architecture design

##### 2.1.1.1 Multi-branch independent encoder

To avoid ambiguity in the mathematical notation, we use m ∈ {d, a, f} to denote the DWI, ADC, and FLAIR branches, respectively, and k to denote the feature scale or encoder stage. The modality-specific feature at scale k is denoted as $F_{k}^{m}$ . The encoder-fused feature, graph-enhanced feature, and final fused feature are denoted as $H_{k}^{e\mathrm{nc}}$ , $H_{k}^{\text{graph}}$ , and $H_{k}^{\text{fused}}$ , respectively. The predicted probability map is denoted as P, and the entropy-based uncertainty map is denoted as U. These symbols are used consistently in the following module descriptions.

Because DWI, ADC, and FLAIR are generated by different imaging mechanisms and may present lesion regions with inconsistent intensity patterns, we did not concatenate the three modalities directly at the input stage. Instead, three parallel encoder branches were used so that each modality could first learn its own early representation before cross-modal fusion was introduced. The input data $X\in {\mathbb{R}}^{1\times D\times H\times W}$ is a single-channel 3D volumetric image. Each encoder branch contains five resolution stages (Stages 0–4), and the number of feature channels is set to {32,64,128,256,512}. Each stage contains two consecutive convolutional blocks:


$$F_{l}^{m}=\phi (\text{IN}(W_{l}^{m}*F_{l-1}^{m}))$$

Here, m ∈ {dwi, adc, flair} denotes the modality branch, and W_l_ denotes the 3 × 3 × 3 convolution kernel. Instance normalization, written as IN(·), was adopted because training was performed with a batch size of 1, under which batch-dependent normalization is usually less stable. The activation function *φ*(·) denotes PReLU, which was used to preserve gradient flow in the negative activation range. Downsampling between adjacent stages was implemented with stride-2 convolutions, thereby producing a multi-scale feature pyramid.

##### 2.1.1.2 Decoder

**Figure 1:**
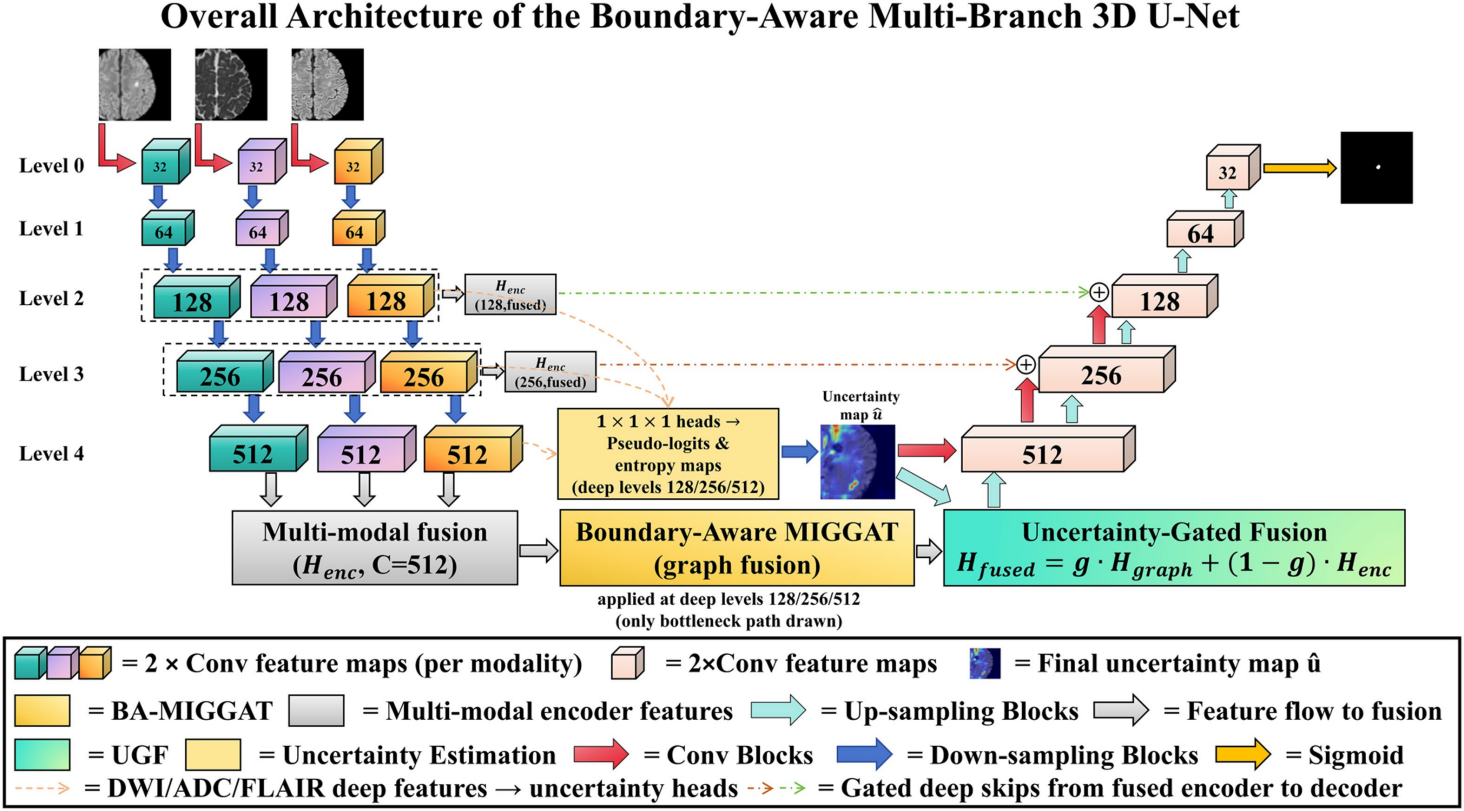
Overall architecture of the proposed MBGS-UNet. The model uses three independent encoder branches to extract modality-specific features from DWI, ADC, and FLAIR. Shallow features are fused by per-modality gating, whereas deeper features are processed by Boundary-Aware MIGGAT and uncertainty-gated fusion. The decoder then reconstructs the lesion probability map through skip connections and progressive upsampling.

The decoder follows a conventional coarse-to-fine reconstruction process. Starting from the bottleneck layer, transposed convolutions are used to progressively recover spatial resolution while reducing the number of channels by half at each stage. At each scale k∈{3,2,1,0}, the upsampled features are concatenated with the corresponding encoder features through skip connections (15, 16) and the fused representations are then refined by two convolution-IN-PReLU residual blocks (17, 18). Finally, a 1 × 1 × 1 convolution head maps the decoded features to logits, and a Sigmoid function is applied to generate the lesion probability map. The full framework of MBGS-UNet is presented in Figure 1.

#### 2.1.2 Hierarchical feature fusion strategy

We designed a hierarchical fusion strategy at skip connections to adapt to feature properties across different resolution levels.

##### 2.1.2.1 Shallow fusion: per-modality gating mechanism (scales 0–1)

At the shallow fusion stage, feature maps retain high-resolution geometric information with strong inter-modality consistency. We employ a lightweight per-modality gating mechanism. For feature $F_{k}^{m}$ , a subnetwork computes weight vector $G_{k}^{m}$ , which is scaled by temperature coefficient τ (set as τ=1.2 in this paper):


$$W_{k}^{m}=\sigma (G_{k}^{m}/\tau )$$

This coefficient is used to smooth the sigmoid output and prevent overly sharp gate responses during the early training stage. The value *τ* = 1.2 was used as a mild temperature-scaling factor for the gating function. This setting was intended to reduce the risk of early gate saturation while still allowing the model to learn spatially varying modality weights. The same value was kept fixed across all experiments. Weighted features are concatenated along the channel dimension and linearly combined via 1×1×1 convolution to generate shallow features $H_{k}^{\text{shallow}}$ .

##### 2.1.2.2 Deep fusion: uncertainty estimation and interaction (scales 2–4)

At the deep stage, features are semantically rich but low in resolution, often exhibiting nonlinear semantic conflicts between modalities. Here we introduce the core modules: Boundary-Aware MIGGAT and uncertainty-gated fusion.

#### 2.1.3 Boundary-aware adaptive graph attention (boundary-aware MIGGAT)

Traditional GAT tends to suppress interactions between highly uncertain features to resist noise (9), leading to loss of information at lesion edges. We propose a boundary-aware mechanism that dynamically adjusts attention strategies based on voxel location.

##### 2.1.3.1 Graph construction and input definition

We treat spatial position p as a subgraph, with node set $V_{p}=\{h_{d},h_{a},h_{f}\}$ . Simultaneously, using the probabilistic graph $P_{k}^{m}$ output by the auxiliary segmentation head, we compute normalized Shannon entropy as the voxel-level uncertainty graph $U_{k}^{m}\in [0,1]$ .

##### 2.1.3.2 Lightweight boundary detector

To achieve spatial adaptive adjustment, we embedded a detector within the GAT. This detector receives multimodal features, extracts edge features through 3×3×3 convolution, and finally outputs a Boundary Probability Map $P_{\mathrm{bnd}}\in (0,1)$ via Sigmoid as a soft switch for subsequent computations.

##### 2.1.3.3 Boundary-adaptive attention calculation

This forms the core of this module. We introduce an adaptive reward-punishment term $\Omega_{\mathrm{ij}}$ modulated by $P_{\mathrm{bnd}}$ to adjust attention scores. For modalities i and j, we first compute the baseline similarity $S_{\mathrm{ij}}^{\text{base}}$ and uncertainty difference $\Delta u_{\mathrm{ij}}=|u_{i}-u_{j}|$ . The final score $S_{\mathrm{ij}}$ is defined as:


$$S_{\mathrm{ij}}=S_{\mathrm{ij}}^{\text{base}}+\underset{\Omega_{\mathrm{ij}}}{\underset{︸}{\left[P_{\mathrm{bnd}}⊙(\beta_{b}\cdot \Delta u_{\mathrm{ij}})-(1-P_{\mathrm{bnd}})⊙(\beta \cdot \Delta u_{\mathrm{ij}})\right]}}$$

In this implementation, set $\beta =1.0,\beta_{b}=2.0.$

Non-Boundary Regions (P_bnd → 0):

Corresponds to smooth backgrounds. The formula degenerates into subtracting $\beta \cdot \Delta u_{\mathrm{ij}}$ , i.e., penalizing differences to suppress noise propagation.

Boundary Regions (P_bnd → 1):

Corresponds to lesion edges. The formula transforms into adding $\beta_{b}\cdot \Delta u_{\mathrm{ij}}$ , i.e., rewarding differences, leveraging complementary gradients between modalities (e.g., sharp DWI boundaries and blurred ADC) to sharpen edges.

**Figure 2:**
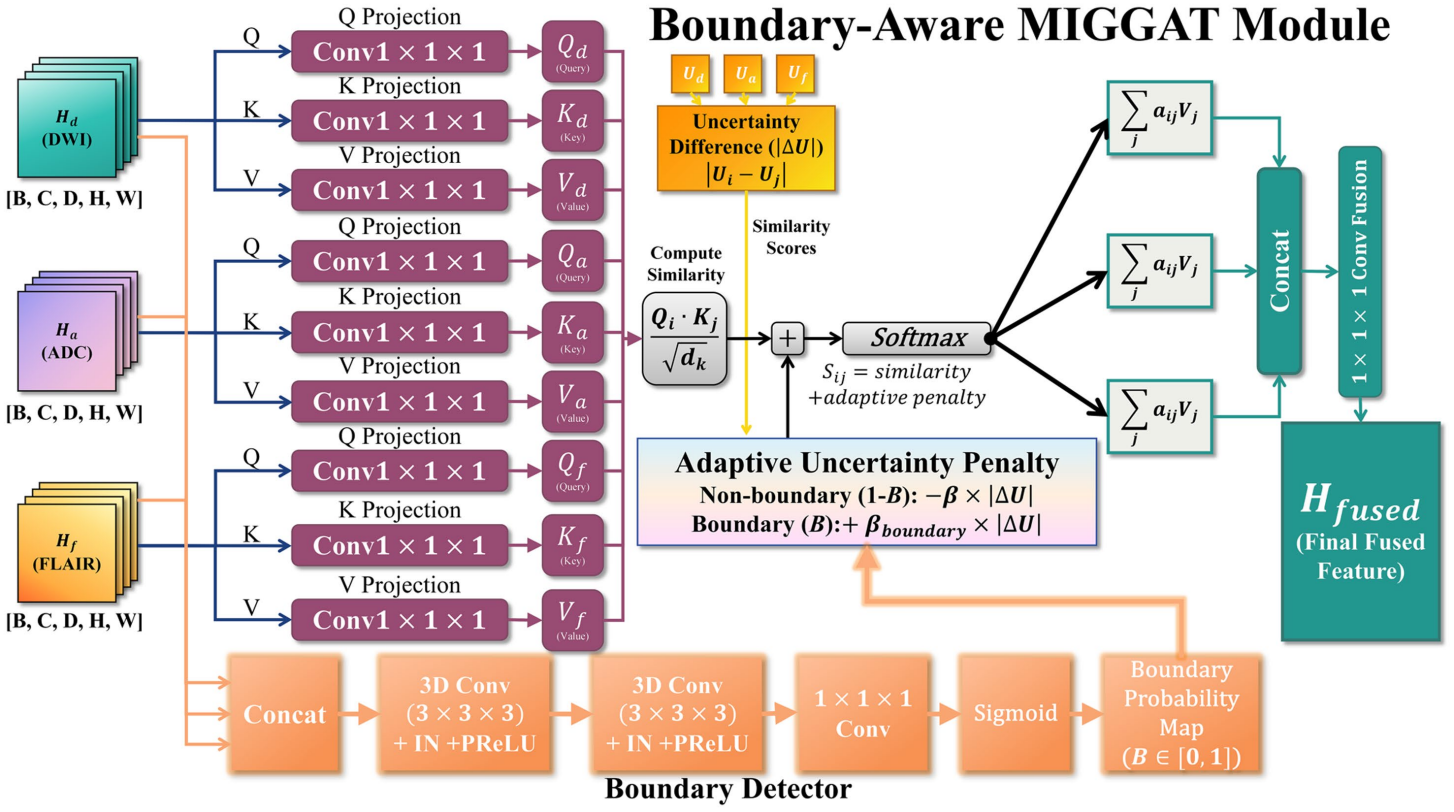
Schematic diagram of the Boundary-Aware MIGGAT module. Each modality is treated as a node in a modality-interaction graph. The boundary probability map is used as a soft switch to penalize uncertainty differences in non-boundary regions and reward complementary intermodal differences in boundary regions. This design aims to suppress noisy propagation while preserving boundary-related information.

The final attention weights are normalized via Softmax, then aggregated to refine the feature map $H_{k}^{\text{graph}}$ . The detailed structure of the boundary-aware MIGGAT module is given in Figure 2.

#### 2.1.4 Uncertainty-gated fusion

The feature $H_{k}^{\text{graph}}$ processed by image attention enhances interactivity but may lose high-frequency textures present in the original encoded feature $H_{k}^{\mathrm{enc}}$ (directly fused via 1×1×1 convolution). To balance correcting feature bias with preserving original image details, we designed an uncertainty gating module. This module utilizes cross-modal average uncertainty $U_{k}^{\mathrm{avg}}=\frac{1}{3}\sum u_{m}$ as prior information. By concatenating $H_{k}^{\text{graph}},H_{k}^{\mathrm{enc}},U_{k}^{\mathrm{avg}}$ and applying convolution with Sigmoid, it generates the spatial gating map $G_{k}(\tau =1.2)$ :


$$G_{k}=\sigma (\text{Conv}([H_{k}^{\text{graph}}∥H_{k}^{\mathrm{enc}}∥U_{k}^{\mathrm{avg}}])/\tau )$$

**Figure 3:**
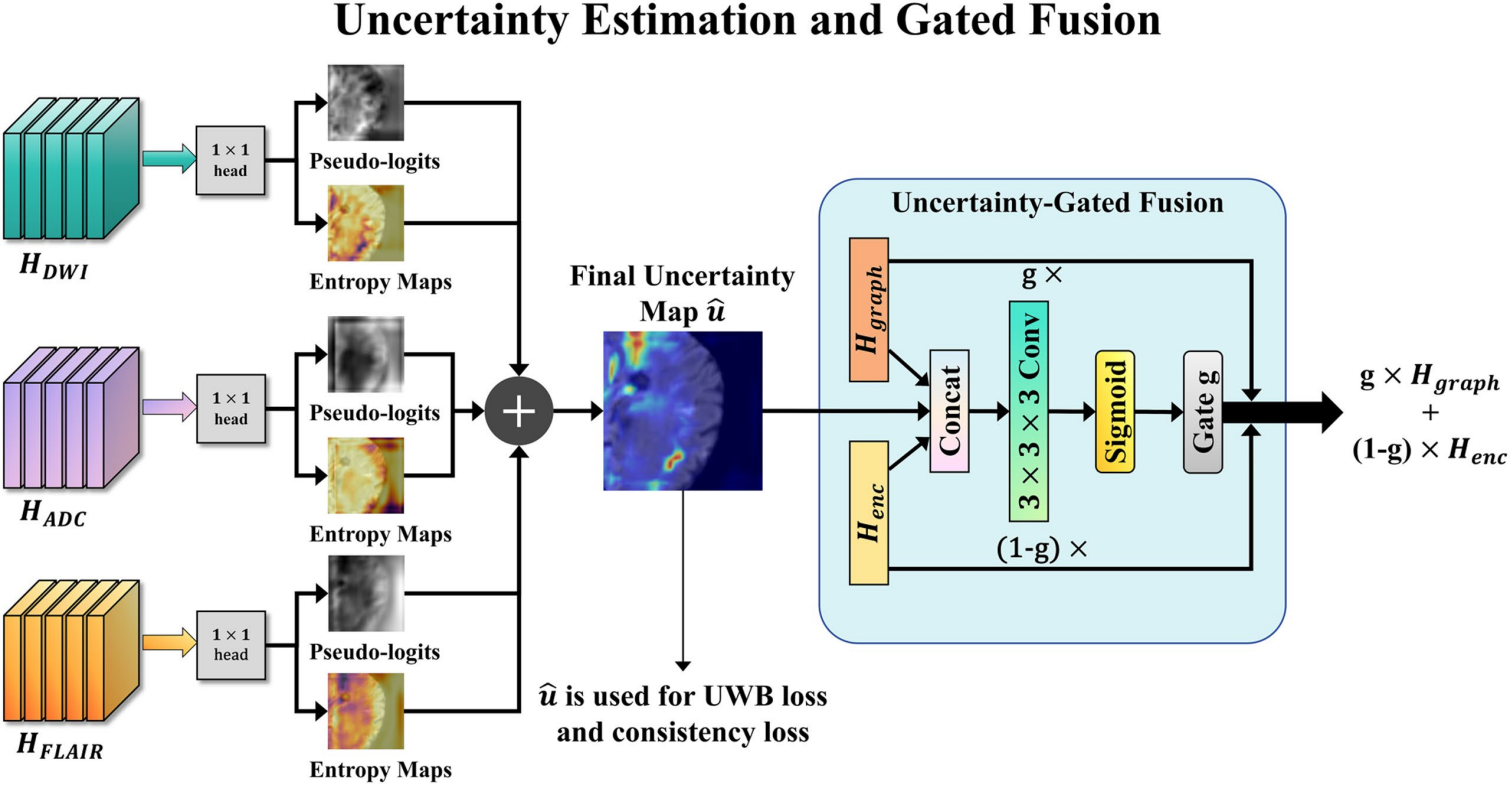
Uncertainty-gated fusion mechanism. The module combines graph-enhanced features with encoder features using a spatial gate generated from graph-enhanced features, encoder features, and the average uncertainty map. Higher gate values indicate greater reliance on graph-enhanced features, whereas lower gate values indicate greater retention of original encoder features.

Final fusion feature: $H_{k}^{\text{fused}}=G_{k}⊙H_{k}^{\text{graph}}+(1-G_{k})⊙H_{k}^{\mathrm{enc}}$ . In regions of high uncertainty, the network automatically increases $G_{k}$ , relying more heavily on graph attention features for refinement. Figure 3 illustrates how uncertainty estimation is coupled with the gating-based fusion process.

#### 2.1.5 Loss functions and training

We designed a hybrid loss function ${ℒ}_{\text{total}}={ℒ}_{\text{main}}+{ℒ}_{\mathrm{UWB}}+\lambda_{\mathrm{reg}}{ℒ}_{\mathrm{reg}}$ and introduced a consistency learning strategy.

##### 2.1.5.1 Uncertainty-weighted boundary loss (UWB loss)

To place more emphasis on difficult voxels, especially those around ambiguous lesion boundaries, the proposed UWB loss dynamically reweights the voxel-wise BCE term (19).

###### 2.1.5.1.1 Boundary ring extraction

Boundary voxels are extracted by a simple morphological operation. In the code, 3D max pooling (*k* = 3) simulates dilation and erosion to compute the boundary ring B=Pool(Y)−(−Pool(−Y)) .

###### 2.1.5.1.2 Dynamic weighting

Combining cross-modal prediction variance $V_{\mathrm{unc}}$ , the voxel weight W is defined as:


$$W=1+\lambda_{\mathrm{bnd}}\cdot {\left(P-0.5\right)}^{2}⊙V_{\mathrm{unc}}⊙B$$

here, $\lambda_{\mathrm{bnd}}$ is set to 0.4. This term mainly increases the penalty on boundary voxels where the prediction appears confident but disagreement across modalities is still present.

##### 2.1.5.2 Uncertainty-guided consistency learning

To improve robustness under perturbation, we further introduced a teacher-student framework (20).

###### 2.1.5.2.1 Weak-strength perturbation

The student branch receives perturbed inputs generated by random scaling with a scale factor of 0.9–1.1, bias perturbation of −0.05 to 0.05, and Gaussian noise with *σ* = 0.03.

###### 2.1.5.2.2 High-uncertainty region filtering

The consistency constraint is imposed only on regions that are both highly uncertain and close to lesion boundaries. Based on the teacher uncertainty map U_map_, the final mask is defined as $M_{\text{final}}=I(U_{\mathrm{map}}>0.6)⊙B$ .

###### 2.1.5.2.3 Consistency loss

The Kullback–Leibler divergence between the teacher and student predictions is computed only within the masked region $M_{\text{final}}$ . The weight follows a Sigmoid ramp-up strategy, gradually increasing to 0.1 over the first 20 epochs. This allows the model to learn foundational features first before introducing consistency constraints.

### 2.2 Dataset and preprocessing

We used the ISLES-2022 multimodal stroke lesion segmentation dataset (4), which includes DWI, ADC, and FLAIR images together with corresponding lesion annotations. To reduce inter-case variation in the raw data, all cases first underwent common preprocessing steps, including bias-field correction, skull stripping, multimodal registration, intensity normalization, and resampling. After these common steps, different strategies were used for model development and independent test-set inference. The complete preprocessing workflow is provided in Supplementary Figure S4.

1. Bias-field Correction: The N4 algorithm was applied to reduce intensity bias caused by magnetic field inhomogeneity (21);
2. Consistent Skull Stripping: Although the ISLES-2022 dataset provides skull-stripped images, we applied HD-BET as an additional brain-extraction step to improve the consistency of brain masks across DWI, ADC, and FLAIR (22). This step was intended to reduce residual non-brain structures, such as eyeballs and skull fragments, that could affect subsequent registration and model training.
3. Multimodal Registration: Using DWI as the fixed template, ADC and FLAIR images were aligned to the same anatomical space via ANTs SyN nonlinear registration (23);
4. Intensity Normalization: Z-score normalization was performed within brain tissue masks, with gray-scale ranges unified;
5. Resampling, model-development patch extraction, and independent test-set inference: After registration and intensity normalization, the multimodal images were resampled to 1 mm^3^ isotropic resolution. For model development, fixed-size 128 × 128 × 128 patches were extracted from the training and validation cases to standardize the network input size and reduce GPU memory consumption. During this stage, lesion annotations were used only to define lesion-centered patches and to compute the supervised segmentation loss. The lesion mask was not used as an image input channel.

For the independent test set, lesion annotations were not used for ROI localization or input-window selection. Each test case was evaluated using label-free sliding-window inference with a window size of 128 × 128 × 128 and an overlap of 0.5. The fivefold-specific models generated probability maps over the inference region, and the final probability map was obtained by averaging the five outputs before applying the validation-derived threshold. The test-set lesion annotations were used only after prediction to compute Dice, HD95, ASSD, and other evaluation metrics.

**Figure 4:**
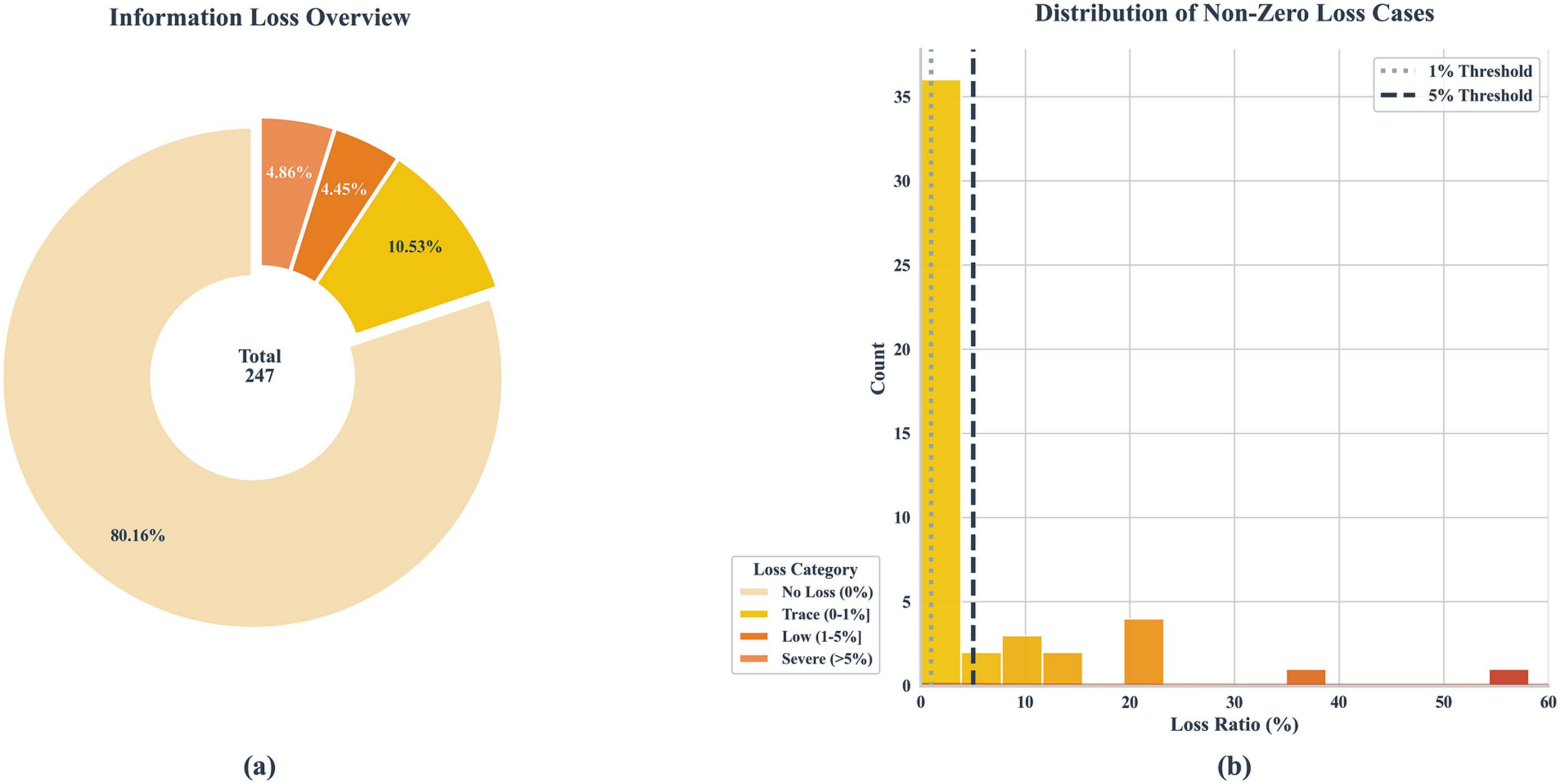
Retrospective analysis of lesion information loss under fixed-size lesion-centered patch extraction. **(a)** Proportions of the 247 valid labeled cases in the four predefined lesion-information-loss categories. **(b)** Distribution of the non-zero lesion-information-loss ratios; the dashed vertical lines indicate the 1% and 5% thresholds used to define the loss categories. This analysis assessed whether a 128 × 128 × 128 patch could preserve most lesion tissue under a retrospective patch setting. It was not part of label-free independent test-set inference.

In addition, we performed a retrospective dataset-level lesion-preservation analysis on the 247 valid labeled cases to characterize whether a fixed 128 × 128 × 128 lesion-centered patch could preserve most lesion tissue under a retrospective patch setting. This analysis was used only to describe the spatial coverage of the patch size and was not used to define the inference region for the independent test set. A quantitative summary of this lesion-preservation analysis is provided in Table 1, and the case distribution of lesion information loss is presented in Figure 4.

**Table 1:** Retrospective dataset-level analysis of lesion preservation under fixed 128 × 128 × 128 lesion-centered patch extraction.

Total sample count		250	
Number of empty lesion samples		3	
Valid sample count		247	
Category	Quantity	Percentage	Cumulative percentage
No loss (0%)	198	80.16%	80.16%
Minimal loss (0% ~ 1%)	26	10.53%	90.69%
Moderate loss (1% ~ 5%)	11	4.45%	95.14%
Severe loss (>5%)	12	4.86%	100.00%

Statistics were calculated on the 247 valid labeled cases only to characterize the spatial coverage of the fixed patch size under a retrospective setting. This analysis was not used to define the inference region for the independent test set, and test-set lesion annotations were used only after prediction for metric calculation.

#### 2.2.1 Dataset splitting and 5-fold cross-validation

The public ISLES-2022 dataset contains 250 cases. To keep model development and final evaluation clearly separated, we used the following data partition strategy:

#### 2.2.1.1 Independent test set

Fifty cases were randomly reserved as a completely independent test set and were used only for the final evaluation. During independent test-set inference, lesion annotations were not used for ROI localization or input window selection; they were used only after prediction for quantitative metric calculation.

#### 2.2.1.2 5-fold cross-validation

**Figure 5:**
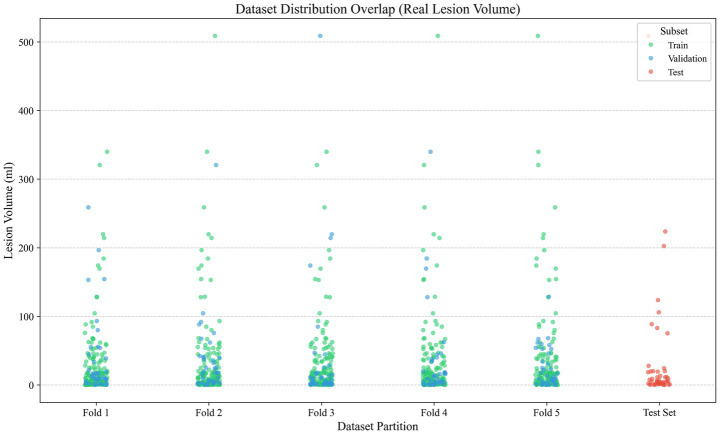
Lesion volume distribution across datasets. Distribution of lesion volumes across the training, validation, and test sets. Green, blue, and red points denote the training, validation, and test subsets, respectively. The three subsets show substantial overlap in lesion-volume range.

The remaining 200 cases were used for model development. After data cleaning, three cases with missing annotations (Cases 150, 151, and 170) were excluded, and five-fold cross-validation was carried out on the final 197 valid cases. Model performance on the independent test set was obtained by ensemble inference from the fivefold-specific models (24). The reported final result therefore represents the average prediction of the five cross-validated models on the test set. The lesion volume distributions of the training, validation, and test subsets are shown in Figure 5.

### 2.3 Implementation details

The model was implemented in PyTorch and MONAI frameworks (25), and all experiments were conducted on a single NVIDIA RTX5090 (32GB) GPU.

During training, online 3D augmentation was applied, including random flipping, random 90° rotation, mild affine transformation, Gaussian noise, and contrast perturbation. Optimization was performed with AdamW using an initial learning rate of $1\times 10^{-4}$ , weight decay $1\times 10^{-5}$ , and the learning rate was adjusted with ReduceLROnPlateau according to the validation Dice score (26). Mixed-precision training (AMP) was enabled throughout the training process to reduce GPU memory consumption and improve convergence efficiency. Representative multimodal inputs and augmentation examples are provided in Supplementary Figure S5.

To provide a clearer description of the experimental setup, we summarize the main training and inference parameters here, with the complete hyperparameter configuration listed in Supplementary Table S3. A deterministic seed of 42 was used to reduce random variation across experiments. During training, the network input was a three-channel 3D patch of size 3 × 128 × 128 × 128, and the batch size was set to 1. The model was trained for up to 120 epochs, with validation performed every 5 epochs. The initial learning rate was 1 × 10^−4^. AdamW was used for optimization, with a weight decay of 1 × 10^−5^ for the main network parameters. Parameters related to the gating modules were trained with a smaller learning rate, set to 0.1 times the main learning rate, and no weight decay was applied to these parameters. The learning rate was adjusted using ReduceLROnPlateau, with a reduction factor of 0.5, a patience of 3, and a minimum learning rate of 1 × 10^−6^. Training was stopped early if the validation Dice did not improve for 10 consecutive validation rounds. Mixed precision training was used, and gradient clipping with a maximum norm of 5.0 was applied to reduce occasional gradient instability.

For the Boundary-Aware MIGGAT module, we used a single head modality-interaction attention design over the three modality nodes, corresponding to DWI, ADC, and FLAIR. The attention projection ratio was set to 0.5, the uncertainty penalty coefficient β was set to 1.0, and the boundary enhancement coefficient was set to 2.0. During inference, the best model from each of the five cross-validation folds was loaded. During independent test-set inference, lesion annotations were not used to define the inference region or select input windows. Each model produced a probability map using sliding-window inference with a window size of 128 × 128 × 128, a sliding-window batch size of 1, and an overlap of 0.5. The final probability map was obtained by averaging the five probability maps and then applying the validation-derived threshold.

### 2.4 Evaluation metrics

To evaluate both segmentation accuracy and the reliability of uncertainty estimation, we used the following groups of metrics:

#### 2.4.1 Conventional segmentation metrics

##### 2.4.1.1 Dice similarity coefficient

DSC measures the overlap between the predicted probability map P and the ground-truth label Y, and it was used as the main metric for overall segmentation accuracy (27).

##### 2.4.1.2 95% Hausdorff distance and average surface distance

These two metrics were used to evaluate boundary-level geometric agreement between the prediction and the reference annotation. HD95 reflects the 95th percentile of the maximum boundary distance and is less sensitive to extreme outliers, making it useful for assessing segmentation quality near lesion margins, especially in irregular regions (23, 27, 28).

#### 2.4.2 Uncertainty-weighted accuracy

Because stroke lesions often have indistinct boundaries and are difficult to segment consistently, we further introduced the uncertainty-weighted accuracy metric. This metric is intended to reflect model behavior in highly uncertain regions, where prediction errors are more likely to occur.

Specifically, we first computed voxel-level prediction entropy based on the probability map from the final segmentation head output to generate an uncertainty map. We then selected the voxel set $\Omega_{K}$ with the highest uncertainty values. The binary prediction at voxel i was defined as ${\hat{Y}}_{i}=I(P_{i}\geq t)$ , where P_i_ denotes the predicted lesion probability and t denotes the binarization threshold used in the corresponding evaluation setting. UWA@TopK was defined as the voxel-level classification accuracy within $\Omega_{K}$ :


$${\hat{Y}}_{i}=I(P_{i}\geq t)$$

$$\mathrm{UWA}@\mathrm{Top}K=\frac{1}{|\Omega_{K}|}\sum_{i\in \Omega_{K}}I({\hat{Y}}_{i}=Y_{i})$$

Here, I(⋅) denotes the indicator function. Y_i_ denotes the ground-truth label at voxel i, and $\Omega_{K}$ denotes the set of voxels with the top K% highest uncertainty values. In this experiment, K ∈ {5, 10, 20} was evaluated, and UWA@Top20% was used as the main reported uncertainty-sensitive metric. A higher UWA value indicates that the model maintains more accurate voxel-level predictions in high-uncertainty regions, such as fuzzy lesion boundaries.

#### 2.4.3 Brier score

To further assess the quality of probabilistic predictions, we additionally report the whole-volume Brier score as a supplementary calibration-related metric. The Brier score measures the mean squared difference between the predicted lesion probability and the binary ground-truth label over all voxels. A lower Brier score indicates better overall probabilistic accuracy. In this study, the Brier score was computed directly from the pre-threshold probability maps on the independent test set, without post-processing, and was used as a supplementary analysis rather than a primary endpoint.

### 2.5 Statistical analysis

Because all methods were tested on the same independent test set, statistical comparisons were performed in a paired manner between MBGS-UNet and the two main competing methods, M2FNet and UMamba. Dice was treated as the primary metric, whereas HD95 and ASSD were analyzed as secondary geometric metrics. For each metric, two-sided Wilcoxon signed-rank tests were conducted across the 50 test cases. To further describe the uncertainty of the paired effect, 95% bootstrap confidence intervals were estimated from 10,000 resamples of the median paired difference. To account for multiple predefined pairwise comparisons, Holm correction was applied, and a corrected *p* value below 0.05 was considered statistically significant.

## 3 Results

### 3.1 Quantitative results

To systematically evaluate the performance of the proposed MBGS-UNet in multimodal stroke lesion segmentation, we selected eight representative 3D segmentation networks from recent years as comparison methods. These include convolutional-based networks SegResNet, Attention UNet, UX-Net, and MedNeXt; visual Transformer-based SwinUNETR; state-space modeling-based networks, including UMamba and SegMamba; and M2FNet designed for medical scenarios (29–36).

Paired statistical analysis on the independent test set showed that the Dice improvement over M2FNet did not remain statistically significant after Holm correction for multiple comparisons. In addition, no statistically significant differences were found between MBGS-UNet and UMamba for Dice, HD95, or ASSD. These results indicate that the observed performance gain should be regarded as a favorable numerical trend rather than conclusive evidence of superiority.

All models were trained from scratch using the same model-development protocol and evaluated on the independent test set using label-free sliding-window inference. The reported test-set metrics were computed after prediction, and test-set lesion annotations were used only for quantitative metric calculation. Evaluation metrics include Dice, HD95, ASSD, and UWA@Top20%, alongside reporting parameters (Params) and computational complexity (MACs). Quantitative results are presented in Table 2, and case-wise paired statistical comparisons against the two main competing models are reported in Table 3.

**Table 2:** Quantitative comparison results of different 3D segmentation models on the ISLES-2022 acute ischemic stroke dataset.

Models	Dice	HD95(mm)	ASSD(mm)	UWA@Top20%	Params(M)	MACs(G)
SegResNet	0.6968	12.92	**2.72**	0.6354	18.970	612.794
SwinUNETR	0.6693	13.92	2.93	0.6108	62.190	786.448
Attention UNet	0.6483	15.37	3.51	0.6078	23.628	584.409
UX-Net	0.6620	22.27	4.89	0.6111	**9.423**	577.776
UMamba	0.7016	17.54	3.88	0.6416	32.140	2338.966
M2FNet	0.7068	13.36	3.64	**0.6461**	51.886	828.012
SegMamba	0.6923	13.36	3.55	0.6424	66.886	1565.714
MedNeXt	0.6836	**11.90**	2.92	0.6381	10.616	**255.861**
Ours	**0.7208**	12.57	2.95	0.6397	68.542	874.878

Dice, HD95, and ASSD, respectively, reflect overall overlap accuracy and boundary error, while UWA@Top20% measures prediction accuracy on high-uncertainty voxels. Higher Dice and UWA values indicate better performance, whereas lower HD95 and ASSD values indicate better boundary agreement. Params denotes model parameter count, and MACs indicates patch-level computational complexity. MACs were computed for one 3D input patch of size 3 × 128 × 128 × 128, rather than full-volume sliding-window inference, to provide a standardized basis for comparing model complexity. For Params and MACs, lower values indicate lower model complexity. Among the numerical entries, bold indicates the best value for each metric, and underlining indicates the second-best value. The proposed method obtained the highest numerical Dice score in this experiment, while showing competitive boundary-related and uncertainty-related performance under the same evaluation protocol.

**Table 3:** Case-wise paired statistical comparison between MBGS-UNet and the main competing models on the independent test set.

Comparison	Metric	Median paired difference	95% CI	Raw p	Adjusted p
Ours vs. M2FNet	Dice	0.0075	[0.0021, 0.0163]	0.0107	0.0644
Ours vs. M2FNet	HD95	0.0000	[−0.3843, 0.0000]	0.1845	0.4757
Ours vs. M2FNet	ASSD	−0.0320	[−0.0863, 0.0231]	0.1571	0.4757
Ours vs. UMamba	Dice	0.0006	[−0.0083, 0.0077]	0.7091	0.7091
Ours vs. UMamba	HD95	0.0000	[−0.9596, 0.1786]	0.1137	0.4757
Ours vs. UMamba	ASSD	−0.0432	[−0.1377, 0.0433]	0.0951	0.4757

Median paired differences are reported as MBGS-UNet minus comparator. Positive values indicate better performance for Dice, whereas negative values indicate better performance for HD95 and ASSD. Corrected *p*-values were obtained using the Holm procedure.

In terms of overall overlap accuracy, MBGS-UNet achieved a mean Dice score of 0.7208 on the independent test set, which was the highest numerical value among the compared methods in this experiment. Its mean Dice was higher than that of M2FNet (0.7068) and UMamba (0.7016), but this numerical advantage should be interpreted together with the paired statistical analysis reported in Table 3. By comparison, the more conventional CNN-based models, including SegResNet, Attention UNet, and UX-Net, all remained below 0.70, while SwinUNETR reached 0.6693. These results suggest that multimodal stroke lesion segmentation on ISLES-2022 remains challenging when modality heterogeneity and boundary ambiguity are present at the same time. Under this setting, MBGS-UNet showed a modest but consistent numerical advantage in Dice.

For boundary-related geometric metrics, the best HD95 was obtained by MedNeXt (11.90 mm), whereas the lowest ASSD was achieved by SegResNet (2.72 mm), indicating that different architectures emphasized different aspects of boundary delineation. MBGS-UNet obtained an HD95 of 12.57 mm and an ASSD of 2.95 mm. Although these values were not the best among all models, they remained competitive and were observed together with the highest numerical Dice value in this experiment. In contrast, SegMamba produced an HD95 of 13.36 mm and an ASSD of 3.55 mm, which were both weaker than those of MBGS-UNet. Overall, these results suggest that the proposed model provides a reasonable balance between lesion overlap and boundary quality.

As a supplementary assessment of probabilistic prediction quality, we calculated the whole-volume Brier score using the raw probability maps before thresholding and post-processing. MBGS-UNet achieved a Brier score of 0.00443, compared with 0.00449 for M2FNet and 0.00510 for UMamba. These values suggest that the probabilistic output of MBGS-UNet was comparable to those of the strongest baselines at the whole-volume level, although this analysis does not establish clinical safety or full calibration performance. Detailed descriptive statistics for the whole-volume Brier scores of MBGS-UNet, M2FNet, and UMamba are provided in Supplementary Table S1.

For the uncertainty-sensitive metric UWA@Top20%, M2FNet, SegMamba, and UMamba achieved values of 0.6461, 0.6424, and 0.6416, respectively, whereas MBGS-UNet reached 0.6397. Although this value was slightly lower than those of the three methods above, it remained higher than that of MedNeXt (0.6381) and clearly exceeded those of SegResNet (0.6354), SwinUNETR (0.6108), and Attention UNet (0.6078). This pattern suggests that the main quantitative advantage of MBGS-UNet lies more in overall Dice performance than in UWA dominance, while still maintaining competitive behavior in uncertain regions.

In terms of computational cost, MBGS-UNet contained 68.542 million parameters and required 874.878 G MACs for one 3D input patch of size 3 × 128 × 128 × 128. By comparison, MedNeXt was much lighter, with 10.616 million parameters and 255.861 G MACs, and it achieved the best HD95 value among all compared models. This indicates that MBGS-UNet should not be regarded as a lightweight architecture.

The increased computational cost mainly comes from the three independent modality encoders, boundary-aware graph interaction, and uncertainty-gated fusion modules. These components were designed to preserve modality-specific information and provide uncertainty-aware interpretation in ambiguous lesion regions, rather than to minimize inference cost. Therefore, the value of MBGS-UNet should be interpreted as a trade-off between competitive segmentation accuracy, uncertainty-aware interpretation, and computational complexity. For resource-constrained clinical environments, lighter models such as MedNeXt may be more suitable when boundary distance and deployment efficiency are the primary priorities.

Additional uncertainty reliability analysis was performed on the independent test set to further examine whether the uncertainty estimates were related to prediction reliability. In the candidate lesion region, the ECE was 0.1343. This result was used as a supplementary calibration-related reference for characterizing the probability maps, rather than as a primary performance endpoint.

The uncertainty estimates showed clear risk-stratification behavior. In the risk-coverage analysis, the voxel-wise error rate was 0.2557 at full coverage, 0.2112 at 80% coverage, and 0.1517 at 50% coverage, corresponding to relative risk reductions of 17.37 and 40.65%, respectively. In addition, case-wise mean predictive entropy was positively correlated with segmentation error, defined as 1-Dice, with a Spearman correlation coefficient of 0.580 (*p* = 1.02 × 10^−5^). These findings suggest that higher uncertainty was associated with less reliable segmentation results and may help identify cases or regions requiring closer review. The corresponding reliability diagram, risk-coverage curve, and uncertainty-error correlation plot are provided in Supplementary Figures S6–S8.

### 3.2 Ablation studies

To assess the contribution of each module and the influence of different architectural design choices, we conducted ablation studies at two levels on the ISLES-2022 dataset. First, we used an incremental approach to estimate the contribution of each core component. Second, we compared several architectural variants under the same evaluation setting. Quantitative results are summarized in Table 4.

**Table 4:** Quantitative results of ablation experiments on the ISLES-2022 dataset.

Method	Dice	HD95(mm)	ASSD(mm)	UWA@Top20%
Baseline	0.6905	11.74	**2.94**	0.6375
+ multimodal encoder	0.7042	12.83	3.25	0.6354
+ Feature fusion	0.7072	13.75	3.36	0.6524
+ Gated skip connection	0.7044	12.71	3.95	0.6466
+ MIG block	0.7061	12.11	3.17	0.6436
+ GAT block	0.7031	**11.73**	3.06	0.6461
+ Uncertainty-gated fusion	0.7084	12.42	3.03	0.6562
+ UWB loss	0.7174	12.16	3.10	**0.6648**
+ Consistency loss	0.7059	12.98	3.59	0.6389
**Variant A (proposed method)**	**0.7208**	12.57	2.95	0.6397
Variant B	0.7059	13.85	3.26	0.6359
Variant C	0.6983	13.56	3.19	0.6354
Variant D	0.7141	14.45	3.22	0.6325
Variant E	0.7134	14.50	3.09	0.6505

This table shows the performance changes when progressively introducing the core components of the proposed framework and compares different architectural variants under the same main ablation evaluation protocol. Variant A represents the complete method proposed in this study, while Variants B to D examine different configurations of the interaction module and boundary supervision. Variant E examines the effect of the consistency learning strategy. Evaluation metrics include Dice, HD95, ASSD, and UWA@Top20%. Higher Dice and UWA values indicate better performance, whereas lower HD95 and ASSD values indicate better boundary agreement. For this table, UWA was calculated under the main ablation evaluation threshold protocol. Because UWA is computed from thresholded binary predictions within the high-uncertainty voxel set, UWA values in this table are not directly interchangeable with those in the post-processing sensitivity analysis, where validation-derived thresholds were applied. Among the numerical entries, bold indicates the best value for each metric, and underlining indicates the second-best value.

#### 3.2.1 Incremental component analysis

We first use the standard UNet as the baseline model and incrementally introduce the proposed enhancement modules to quantify the marginal gains of each component. To assess the model’s robustness in hard-to-classify regions, we additionally report the uncertainty-weighted accuracy (UWA@Top20%) defined in section 2.4.2 as a supplementary metric.

Variant A used cross-modal variance during training to capture feature conflicts and optimize the boundary-related loss. However, this training signal was not used to define UWA during evaluation. For fair comparison with the baseline and other variants, UWA was calculated for all models using the same definition described in section 2.4.2. Specifically, the top 20% high-uncertainty voxels were selected according to the predictive entropy of the final probability map.

The baseline model directly concatenates multimodal data along the channel dimension into a single encoder, yielding a Dice score of only 0.6905. Introducing multi-branch independent encoders improved the Dice score to 0.7042, suggesting that independent modal feature extraction can reduce feature interference caused by premature fusion. Further incorporating a feature fusion module elevated the Dice score to 0.7072, indicating the usefulness of cross-modal feature integration. Regarding feature interaction, incorporating a modality interaction graph module stabilized Dice at 0.7061 while maintaining a low HD95 (12.11 mm). Subsequently, adding a graph attention module further enhanced global context modeling, and reduced HD95 to 11.73 mm. This indicates that the graph structure effectively captures long-range dependencies, improving lesion shape prediction. Following this, introducing an uncertainty-based gated fusion mechanism caused Dice to rebound to 0.7084, suggesting that uncertainty-based feature filtering may reduce noise propagation. Finally, incorporating UWB Loss resulted in a further increase in Dice to 0.7174, suggesting that weighted supervision contributed to improving high uncertainty boundary supervision. Ultimately, integrating a consistency learning strategy alongside all optimizations (i.e., Proposed Method/Variant A) yielded the model’s best overall performance.

#### 3.2.2 Comparative analysis of architectural design choices

After examining the contribution of each individual component, we further compared the final model (Variant A) with several alternative design variants (Variants B, C, D, and E). The purpose of this analysis was to determine whether our choices for the interaction module, boundary supervision, and consistency strategy were reasonable from a quantitative perspective.

We first compared Variant A with Variant B to examine the effect of the interaction module. Variant A uses Boundary-Aware Attention, whereas Variant B relies on hierarchical uncertainty-gated fusion without the same boundary-aware interaction design. Quantitatively, Variant B achieved a Dice score of 0.7059 and an HD95 of 13.85 mm, both of which were inferior to those of Variant A. This comparison suggests that filtering features only at the fusion endpoint is not sufficient. In contrast, Variant A introduces boundary-aware interaction earlier in the propagation process through MIGGAT, which helps preserve complementary edge information across modalities from a relatively early stage.

We next examined the effect of different boundary supervision strategies. Variant C, which used a multi-scale boundary loss derived from Signed Distance Transform, produced the lowest Dice score among all variants (0.6983). This result implies that rigid geometric distance constraints may not be well suited to stroke lesions with indistinct boundaries, where the target shape is often irregular and difficult to optimize directly. Variant D combined UWB Loss with Multi-Scale Boundary Loss. Although its Dice score increased to 0.7141, its HD95 rose to 14.45 mm, which was even worse than that of Variant B. Taken together, these results suggest that directly combining uncertainty-based soft weighting with geometry-based hard constraints did not provide a clear benefit in this setting and instead made optimization more difficult.

We also compared the full model (Variant A) with Variant E to assess the contribution of consistency learning. Relative to Variant E, Variant A reduced HD95 from 14.50 to 12.57 mm and ASSD from 3.09 to 2.95 mm. These changes suggest that the consistency strategy improved boundary regularity and helped stabilize the predicted lesion shape under perturbation. At the same time, Variant E showed a slightly higher UWA@Top20% value than Variant A (0.6505 vs. 0.6397). This pattern points to a trade-off: without the consistency constraint, the model may behave more aggressively in highly uncertain regions, which can occasionally improve local voxel matching, but may also reduce structural stability. In contrast, Variant A appears to sacrifice a small amount of voxel-level performance in exchange for better anatomical consistency. From the overall results in Table 4, this trade-off still supports the choice of Variant A as the final model.

### 3.3 Post-processing strategy

To examine the influence of post-processing under a fixed evaluation protocol, we performed a sensitivity analysis on the three representative variants selected from the ablation experiments. For each selected variant, the binarization threshold was first determined on the validation folds and then fixed before independent test-set evaluation. The validation-derived thresholds were 0.45 for Variant A, 0.45 for Variant D, and 0.40 for Variant E. Because UWA depends on the thresholded binary prediction, UWA values in the post-processing sensitivity analysis may differ from those reported in the main ablation table when different binarization thresholds are used. The candidate threshold grid ranged from 0.30 to 0.70 with a step size of 0.05, and the threshold was selected only on the validation folds. This validation-based procedure was used to avoid selecting thresholds from the independent test set.

After the validation-derived thresholds were fixed, several predefined post-processing settings were evaluated, including connected-component filtering, hole filling, and lightweight morphological smoothing. These settings were intended to examine whether simple morphology-based operations could reduce small isolated false-positive components or boundary irregularities. During independent test-set evaluation, the thresholds were kept fixed, and the predefined post-processing settings were applied only for sensitivity analysis. Hole filling was included as one of the predefined operations to reduce closed cavities in the binary prediction masks. Connected component filtering was applied with predefined physical volume thresholds to remove small isolated components. Lightweight morphological smoothing was also evaluated to assess its effect on boundary regularity. The results showed mixed effects across variants and metrics, so post-processing was not treated as a test-set-optimized performance enhancement. Additional details of the predefined post-processing settings are provided in Supplementary Table S2. The post-processing sensitivity analysis under validation-derived thresholds is summarized in Table 5.

**Table 5:** Post-processing sensitivity analysis using validation-derived thresholds.

Experiment	Method	Dice	HD95(mm)	ASSD(mm)	UWA@Top20%
**Variant A (proposed method) (*t* = 0.45)**	No post-processing	0.7208	12.57	**2.95**	0.6397
	Weak morphology	0.7183	13.13	3.32	0.6278
	Intermediate morphology	0.7204	**11.01**	2.99	0.6414
	Strong morphology	0.7174	12.92	3.03	0.6533
	Adaptive morphology	**0.7210**	12.85	3.01	0.6387
Variant D (*t* = 0.45)	No post-processing	0.7141	14.45	3.22	0.6325
	Weak morphology	0.7109	15.57	4.02	0.6188
	Intermediate morphology	0.7129	12.59	3.33	0.6293
	Strong morphology	0.7099	13.31	3.38	0.6350
	Adaptive morphology	0.7142	13.24	3.34	0.6315
Variant E (*t* = 0.40)	No post-processing	0.7129	14.87	3.21	0.7133
	Weak morphology	0.7091	16.38	3.81	0.6945
	Intermediate morphology	0.7124	14.52	3.27	0.7123
	Strong morphology	0.7090	14.46	3.21	**0.7179**
	Adaptive morphology	0.7136	13.99	3.18	0.7155

The binarization thresholds were selected on the validation folds and fixed before independent test-set evaluation. The selected thresholds were 0.45 for Variant A, 0.45 for Variant D, and 0.40 for Variant E. Under each fixed threshold, predefined morphology-based post-processing settings were evaluated on the independent test set to examine the sensitivity of segmentation results to post-processing. The no post-processing row indicates direct evaluation of the thresholded prediction without morphology-based operations. The independent test set was used only for reporting the performance of these fixed settings, not for threshold tuning or final model selection. Because UWA is calculated from thresholded binary predictions within the high-uncertainty voxel set, changes in the binarization threshold can affect UWA values. Therefore, the no post-processing rows in this sensitivity analysis may differ from the corresponding ablation rows in Table 4 when different thresholds are used. Within each variant block, bold indicates the best value for each metric, and underlining indicates the second-best value.

### 3.4 Visual analysis

To examine how the proposed method behaves on representative cases, we further performed a qualitative analysis from five aspects: result-uncertainty-error correspondence, visual comparison with other models, intermediate module responses, three-dimensional morphological consistency, and cross-fold training stability. All examples were taken from the independent test set. The displayed predictions were obtained from the five-fold ensemble, and the uncertainty maps were computed from the predictive entropy of the final probability map.

**Figure 6:**
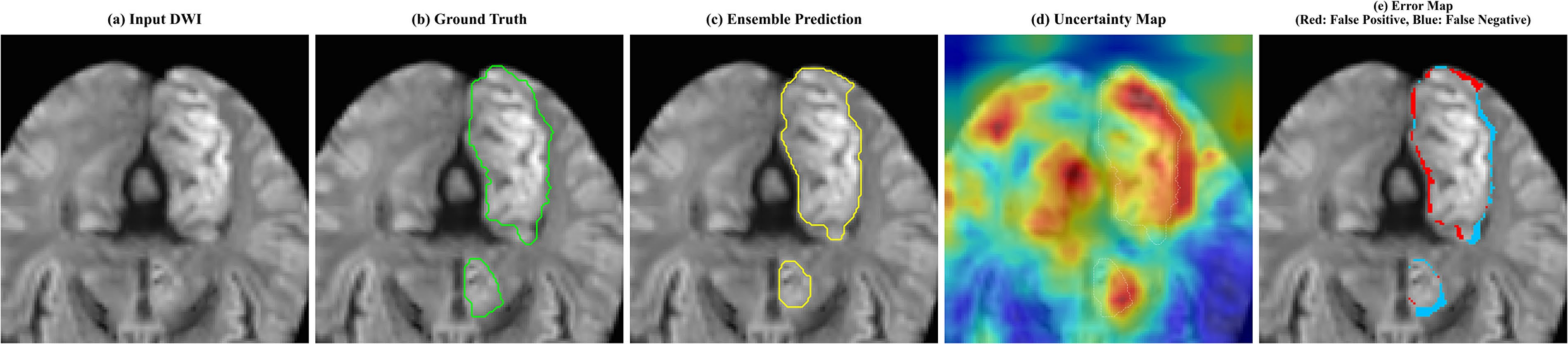
Segmentation result and uncertainty visualization for a representative ISLES case. From left to right are the DWI image, ground-truth annotation, five-fold ensemble prediction, uncertainty heatmap, and error map. In the error map, red indicates false positives and blue indicates false negatives. High-uncertainty responses are mainly distributed near lesion boundaries, where most visible errors also occur.

Figure 6 shows that the five-fold ensemble prediction agrees well with the ground-truth annotation in the major lesion region. At the same time, stronger uncertainty responses are mainly observed near lesion boundaries, morphological transition zones, and other low-contrast areas. In the error map (red for false positives and blue for false negatives), most visible errors are also concentrated in these regions, especially along thin boundary bands and around small discrete lesions. This spatial correspondence suggests that the uncertainty map is closely related to local prediction difficulty and may therefore be useful for visual error localization and subsequent review. An additional example showing the spatial correspondence between uncertainty responses and local prediction errors is provided in Supplementary Figure S3.

**Figure 7:**
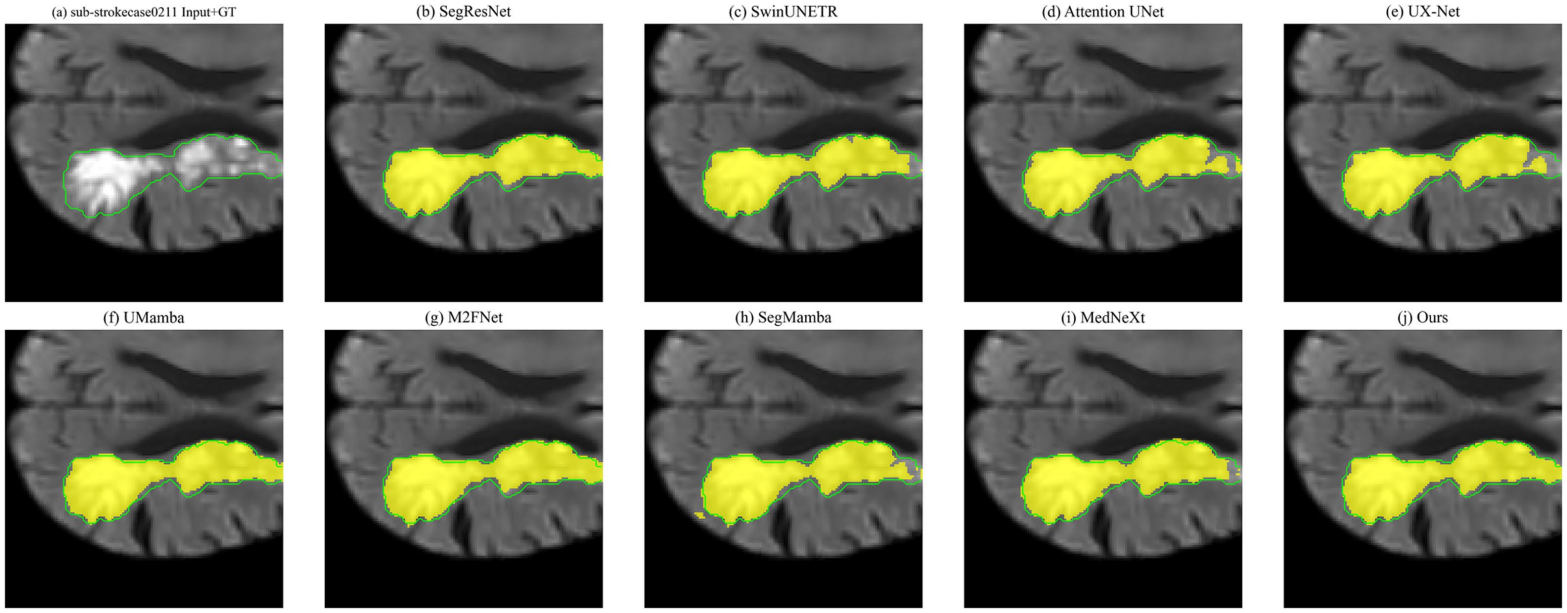
Visual comparison of segmentation results from different models on the same case. **(a)** The input DWI image and ground-truth annotation. **(b–j)** The predictions produced by SegResNet, SwinUNETR, Attention UNet, UX-Net, UMamba, M2FNet, SegMamba, MedNeXt, and MBGS-UNet, respectively. Green contours indicate the reference lesion boundary, and yellow regions indicate the predicted lesion area.

Figure 7 provides a direct visual comparison of different 3D segmentation models on the same case. Most methods are able to cover the major lesion area, but the differences become more obvious near lesion boundaries, where oversmoothing, local omission, or slight over-expansion can still be observed. Compared with the other models, MBGS-UNet preserves the main lesion extent more completely and shows a relatively more stable response for boundary detail and small isolated lesion regions. Its prediction is also visually closer to the reference contour, with fewer obvious false detections in the background. These observations are consistent with the intended role of boundary-aware interaction and uncertainty-guided fusion in reducing noisy cross-modal propagation. Additional qualitative comparisons on two representative ISLES-2022 cases are provided in Supplementary Figures S1 and S2.

**Figure 8:**
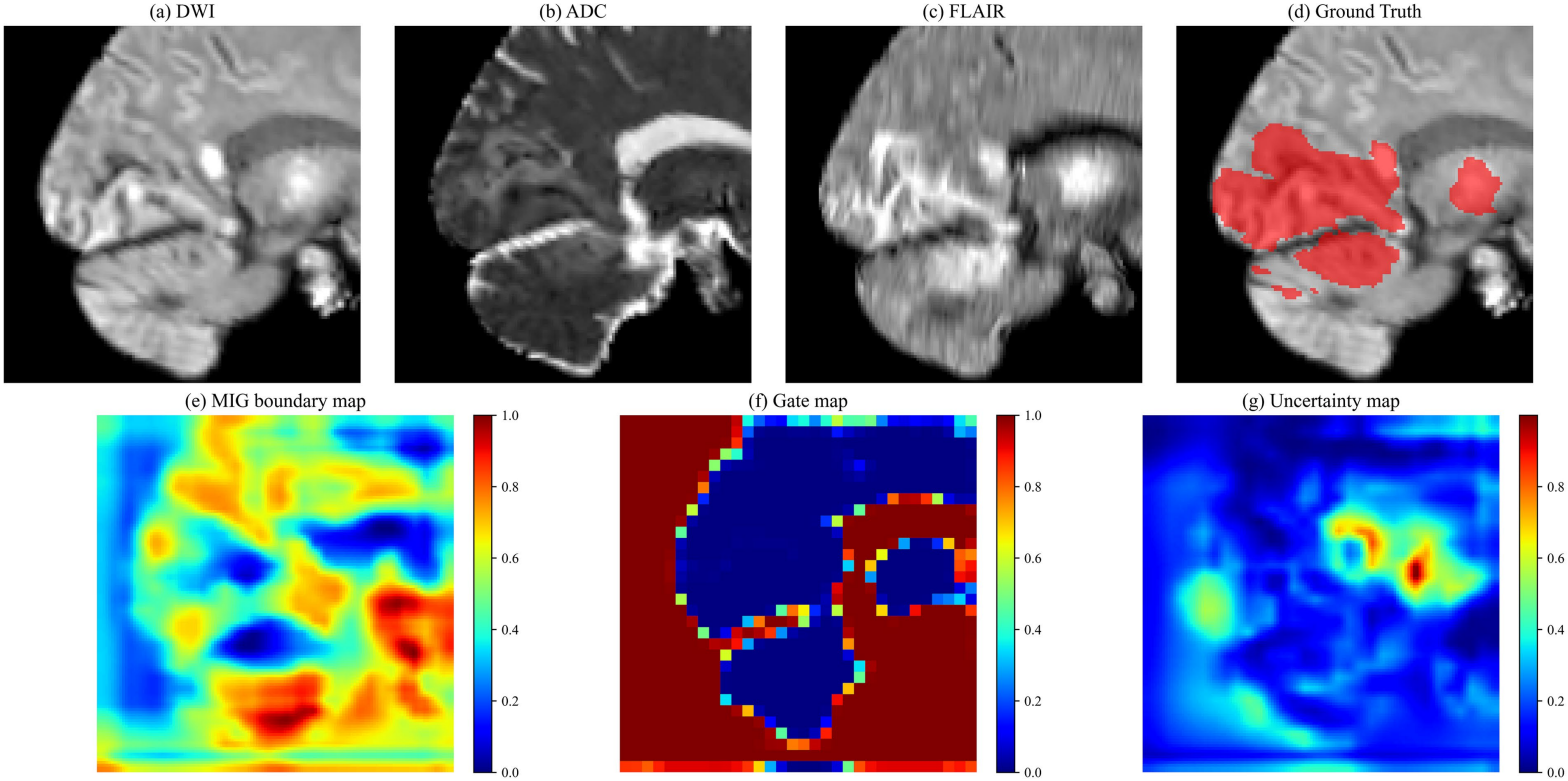
Multimodal images and intermediate response maps from the same case. **(a)** DWI, **(b)** ADC, **(c)** FLAIR, **(d)** ground-truth annotation, **(e)** MIG boundary response map, **(f)** deep gating map, and **(g)** uncertainty map. These panels provide a visual reference for the spatial relationship among lesion location, boundary response, gating intensity, and uncertainty.

Figure 8 further illustrates the relationship between the multimodal inputs and the intermediate response maps. In this example, the MIG boundary map shows stronger activation around lesion contours, suggesting that the boundary-aware interaction module is more responsive in regions where cross-modal disagreement is relatively strong. The corresponding gate map also varies across space rather than remaining uniform, with lower transmission in noisy or ambiguous areas and relatively stronger responses in the core lesion region. In addition, the uncertainty map tends to show higher values near boundaries, and its spatial pattern is broadly consistent with the boundary and gating responses. Taken together, these maps suggest that uncertainty is not only an output for later inspection, but is also closely coupled with the internal fusion behavior of the network.

**Figure 9:**
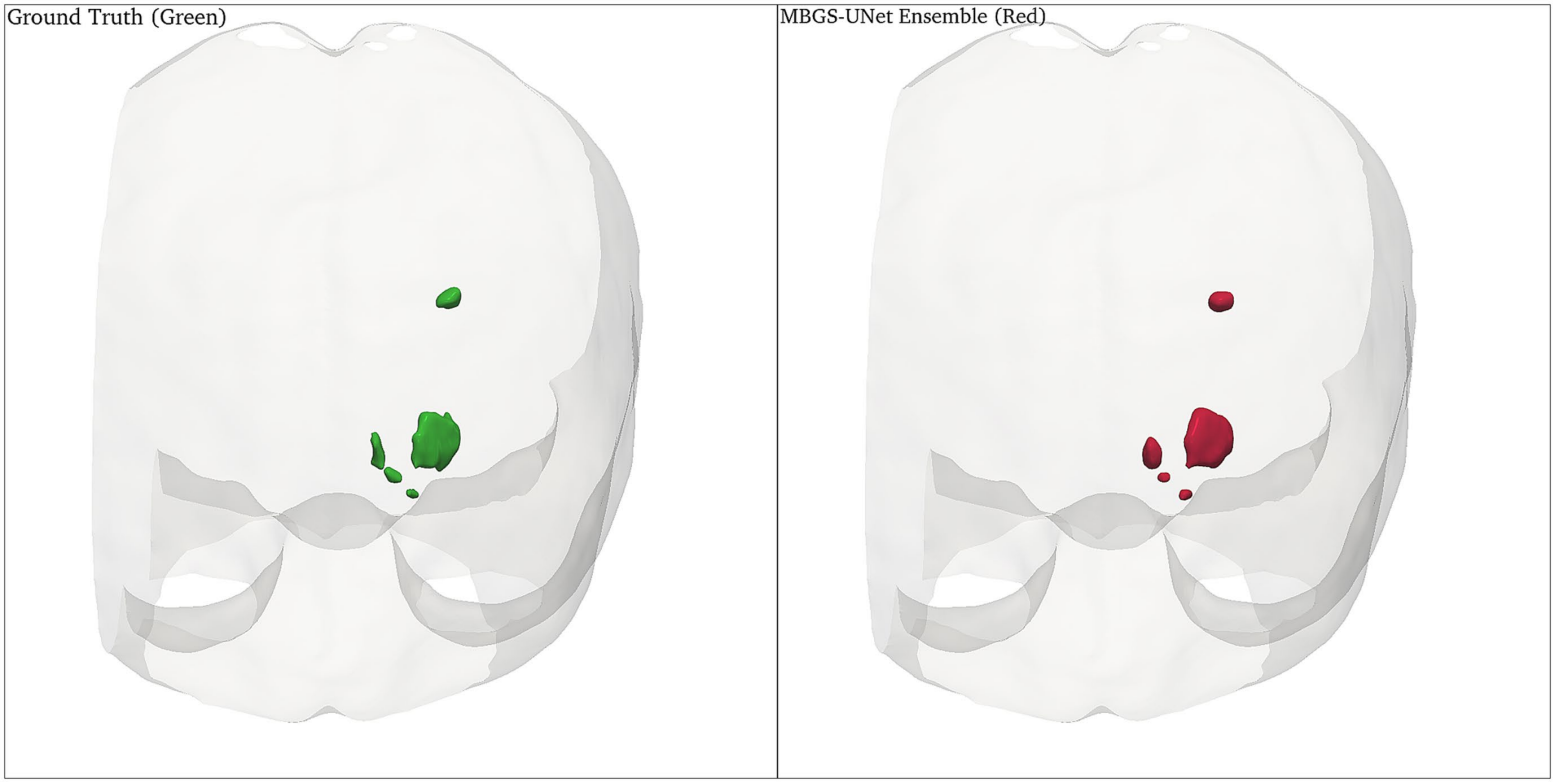
Three-dimensional visualization of lesion morphology. The left panel shows the manually annotated lesion distribution (green), and the right panel shows the three-dimensional prediction of MBGS-UNet obtained from five-fold ensemble inference. The semi-transparent gray structure denotes the brain parenchyma reconstructed from FLAIR images. The predicted lesion distribution is visually close to the reference, with remaining differences mainly located at lesion volume and boundary extent.

Figure 9 shows the three-dimensional relationship between the reference lesion and the MBGS-UNet ensemble prediction. At this level, the predicted lesion distribution is visually close to the annotated lesion in both spatial location and overall morphology, including the main lesion and several discrete components. The remaining differences are relatively limited and mainly appear as small changes in local volume or boundary extent. This pattern is consistent with the slice-level observation that most residual errors are concentrated near difficult boundary regions rather than arising from complete structural mismatch.

**Figure 10:**
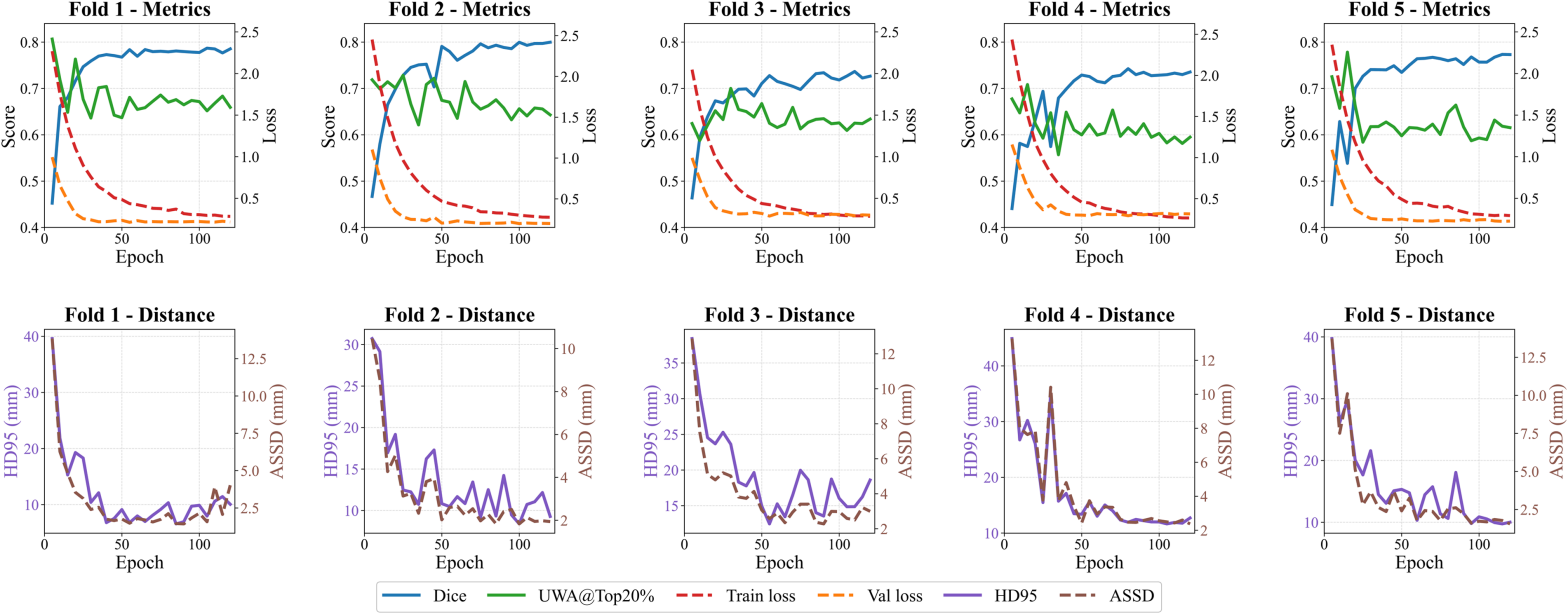
Training curves from five-fold cross-validation. The plots include segmentation metrics (Dice and UWA), loss values, and boundary-related distance metrics (HD95 and ASSD) across training iterations for each fold.

Figure 10 summarizes the training behavior under five-fold cross-validation. Across folds, Dice and UWA generally increased and then became relatively stable as training proceeded, whereas the training and validation losses showed a downward trend. HD95 and ASSD improved more noticeably in the earlier stage and then converged with only limited fluctuation. Although some fold-to-fold variation remained, the overall curve shapes were similar and no obvious divergence was observed. These results suggest that the training process was reasonably stable under the current small-sample and class-imbalanced setting, which also supports the use of fold ensemble inference.

## 4 Discussion

### 4.1 Main findings

This study was designed as an artificial intelligence-assisted approach for cerebrovascular magnetic resonance imaging analysis. The results should not be interpreted as showing definitive superiority in segmentation accuracy, because the paired statistical analysis did not demonstrate significant improvement over the strongest baselines after correction. Instead, the main value of MBGS-UNet lies in combining competitive segmentation performance with uncertainty-aware interpretation. In particular, the uncertainty maps tended to highlight lesion boundaries, small scattered components, and other error-prone regions, suggesting that they may be useful as a review aid rather than as a standalone clinical decision-making tool.

A key feature of MBGS-UNet is that uncertainty is used during fusion rather than only after prediction. In multimodal MRI, boundary regions and low-contrast areas often contain both disagreement and complementarity across modalities. In our model, boundary-aware interaction and uncertainty gating allow the fusion behavior to change with local conditions. In regions with higher uncertainty, the model can make greater use of cross-modal interaction to refine ambiguous responses. In regions with lower uncertainty, it retains more of the original encoded detail. This design helps reduce oversmoothing near lesion borders while preserving fine structural information.

The uncertainty maps may provide practical information beyond visual presentation of the final mask. In our qualitative analysis, stronger uncertainty responses were often observed near lesion boundaries and regions where false positives or false negatives occurred. This suggests that uncertainty maps may help reviewers identify areas where the model prediction is less stable and where closer inspection is needed. Such information does not replace expert judgment, but it may support manual review and multimodal consistency checking in difficult cases.

The additional uncertainty analyses further support this interpretation. The ECE-based reliability analysis provided a supplementary view of probability calibration, while the risk-coverage curve and case-wise uncertainty-error correlation more directly reflected the relationship between uncertainty and prediction reliability. Specifically, retaining lower-uncertainty voxels reduced the voxel-wise error rate, and cases with higher mean predictive entropy tended to have larger segmentation errors. These results suggest that the uncertainty output is more suitable as a risk-stratification and error-localization signal than as a standalone clinical probability for direct decision-making.

The sensitivity of the model to domain shift also needs to be considered. MBGS-UNet uses separate modality encoders and uncertainty-guided interaction, which may help reduce simple feature interference across DWI, ADC, and FLAIR. However, the MIGGAT module still depends on the learned feature distributions, feature similarity, and uncertainty estimates from all three modalities. Therefore, changes in scanner protocol, image contrast, spatial resolution, noise level, or registration quality may affect the attention weights and the final fusion behavior. This issue is especially relevant when one modality is missing or has poor image quality, because the current model was designed for complete three-modality input.

The computational cost of MBGS-UNet should also be considered when interpreting its practical value. Compared with lighter architectures such as MedNeXt, the proposed model requires more parameters and higher patch-level MACs. MedNeXt also achieved the best HD95 value in our comparison, suggesting that lighter models may be preferable when boundary distance and deployment efficiency are the main priorities. Therefore, MBGS-UNet should not be viewed as a universally preferable option for all clinical IT environments.

The higher complexity of MBGS-UNet mainly comes from its multi-branch design and uncertainty-guided interaction modules. The three modality-specific encoders were introduced to preserve DWI-, ADC-, and FLAIR-specific representations before deeper fusion, while the boundary-aware graph interaction and uncertainty-gated fusion modules were designed to provide additional information about ambiguous regions. In this sense, MBGS-UNet represents a trade-off between computational cost, competitive segmentation performance, and uncertainty-aware interpretation.

For deployment, mixed-precision inference may help reduce GPU memory use and improve inference efficiency. However, this does not change the intrinsic parameter count or MACs of the model. Future work should therefore explore lighter variants of the framework, such as model pruning, knowledge distillation, lightweight graph interaction, and modality-adaptive inference, to improve its suitability for resource-constrained clinical environments.

### 4.2 Limitations

Several limitations should be noted. First, the remaining errors were mainly observed in two types of cases: lesions with blurred, elongated, and low-contrast boundaries, and small isolated lesions located away from the main lesion region. These lesions are more vulnerable to local omission, slight over-segmentation, thresholding effects, post-processing variation, and class imbalance. They also often coincided with areas of higher uncertainty, suggesting that uncertainty maps can help reveal difficult regions but cannot remove the underlying segmentation errors.

Second, although independent test-set inference did not use lesion annotations for ROI localization or input-window selection, the model was developed using fixed-size lesion-centered patches during training and validation to reduce GPU memory consumption. This patch-based training strategy helped standardize the input size, but it may not fully match fully automated whole-brain clinical deployment. Future work should investigate whole-brain sampling, brain-mask-based sampling, or more diverse label-free sampling strategies during model development.

Third, the evaluation was based on a public dataset and an internal independent test split. External validation across different centers, scanners, and acquisition protocols was not included. Therefore, the present results do not establish broad generalizability. Domain shift remains a concern because differences in DWI b-values, ADC scaling, FLAIR acquisition, spatial resolution, image noise, and registration quality may affect modality-specific features, uncertainty estimates, and cross-modal attention weights. Although intensity normalization, instance normalization, separate modality encoders, and uncertainty-guided fusion may reduce mild intensity variation, these design choices should not be interpreted as proof of robustness to domain shift.

Fourth, the current model assumes that DWI, ADC, and FLAIR are all available. Because MBGS-UNet explicitly models interactions among the three modality branches, missing or severely degraded modalities may disturb the learned fusion pattern. The model was not trained with modality-dropout, modality-availability masks, or modality imputation. Future work should evaluate these strategies under controlled missing-modality settings and external multi-center data.

Fifth, uncertainty was incorporated into feature fusion and result interpretation, but its practical reliability still requires further validation. To provide a preliminary assessment, we added calibration-oriented analyses, including expected calibration error (ECE) (37), reliability diagrams, uncertainty-based risk-coverage analysis (38), and case-wise uncertainty-error correlation analysis. However, these analyses were retrospective and based on the current independent test set. Therefore, they should be interpreted as supplementary evidence that uncertainty is related to prediction reliability, rather than as proof of clinical safety or calibrated decision-making.

Finally, MBGS-UNet has higher computational cost than several competing architectures, especially MedNeXt. Mixed-precision inference may reduce memory usage and improve inference efficiency, but it does not change the intrinsic parameter count or patch-level MACs. In addition, several module-specific hyperparameters were empirically fixed in the current study, including the gate temperature *τ*, the uncertainty threshold used in consistency learning, the uncertainty penalty coefficient, and the boundary enhancement coefficient in the Boundary-Aware MIGGAT module. The paired statistical analysis also showed that the observed Dice improvement over strong baselines did not remain significant after multiple-comparison correction. Therefore, the current results should be interpreted as a favorable numerical trend rather than conclusive evidence of superiority. Future work should explore lighter variants, hyperparameter sensitivity, model pruning, knowledge distillation, and external prospective validation before clinical deployment.

## 5 Conclusion

In this study, we developed MBGS-UNet as an uncertainty-aware multimodal segmentation framework for acute ischemic stroke lesions. The model was designed to address a practical difficulty in this task: the regions that are most informative for multimodal fusion are often also the regions with the greatest ambiguity and disagreement across modalities. To handle this issue, we combined boundary-aware graph interaction, uncertainty-gated fusion, uncertainty-weighted boundary supervision, and consistency learning within a multi-branch framework.

The experimental results suggest that MBGS-UNet may be useful for artificial intelligence-assisted cerebrovascular image analysis, but its contribution should be interpreted cautiously. Although the model achieved the highest mean Dice score among the compared methods on the independent test set, paired statistical analysis did not demonstrate clear superiority over the strongest baselines after correction for multiple comparisons. Therefore, the main value of the method lies in maintaining competitive segmentation performance while providing uncertainty maps that tend to highlight lesion boundaries and other error-prone regions. These uncertainty outputs may support result interpretation and manual review in difficult cases. Overall, MBGS-UNet should be regarded as an exploratory approach for uncertainty-aware multimodal stroke lesion segmentation, and its broader robustness still requires validation on larger and more diverse external cohorts.

## Data Availability

Publicly available datasets were analyzed in this study. This data can be found at: The datasets analyzed in this study are publicly available from the ISLES 2022 challenge dataset. URL: https://www.isles-challenge.org/.
