# Supplementary material for: Boundary-aware and uncertainty-guided deep learning for multimodal magnetic resonance imaging segmentation of stroke lesions
Source: Front Neurol. 2026 Aug 4;17:1872532. doi: 10.3389/fneur.2026.1872532 (PMC13481876; doi:10.3389/fneur.2026.1872532)
Supplement: Supplementary file 1 [file Data_Sheet_1.pdf]

Supplementary Material

1 Supplementary Data

Supplementary Table S1. Whole-volume Brier score comparison among MBGS-UNet, M2FNet, and UMamba on the independent test set.

Model	mean	std	median	q1	q3	min	max
Ours	0.004430	0.011862	0.001050	0.000467	0.002767	0.000031	0.080306
M2FNet	0.004490	0.011980	0.001034	0.000442	0.003277	0.000040	0.081437
UMamba	0.005097	0.017294	0.000933	0.000415	0.002748	0.000022	0.121589

Supplementary Table S2. Supplementary sensitivity analysis of connected-component filtering under validation-derived thresholds. This table reports the effects of two predefined connected-component filtering strategies with different strengths (Std.Filter and Strong Filter) on three representative model variants (Variants A, D, and E). For each row, the binarization threshold was selected on the validation folds from a predefined grid ranging from 0.30 to 0.70 with a step size of 0.05 and was then fixed before independent test-set evaluation. The independent test set was used only to report the performance of these fixed settings, not for threshold tuning, post-processing selection, or final model selection. The No post-processing row indicates evaluation without connected-component filtering. Dice, HD95, ASSD, and UWA@Top20% are reported. Higher Dice and UWA values indicate better performance, whereas lower HD95 and ASSD values indicate better boundary agreement. UWA values in this supplementary sensitivity analysis were calculated using the validation-derived threshold shown in each row. Since UWA is computed from thresholded binary predictions within high-uncertainty voxels, these values may differ from the ablation results in Table 4 when a different binarization threshold was used. Within each variant block, bold indicates the best value for each metric, and underlining indicates the second-best value.

Experiment	Method	Val-derived threshold	Dice	HD95(mm)	ASSD(mm)	UWA@Top20%
**Variant A (Proposed method)**	No post-processing	0.45	**0.7208**	**12.57**	**2.95**	0.6397
	Std.Filter	0.35	<u>0.7153</u>	16.35	3.44	<u>0.7415</u>
	Strong Filter	0.40	0.7146	<u>14.31</u>	3.39	0.6900
Variant D	No post-processing	0.45	0.7141	14.45	3.22	0.6325
	Std.Filter	0.45	0.7076	15.26	3.69	0.6077
	Strong Filter	0.45	0.7050	14.51	3.69	0.6112

Variant E	No post-processing	0.40	0.7129	14.87	3.21	0.7133
	Std.Filter	0.35	0.7041	17.71	3.66	**0.7455**
	Strong Filter	0.40	0.7037	17.05	3.72	0.6904

Supplementary Table S3. Training and inference hyperparameter settings.

Category	Setting	Value
Random seed	Deterministic seed	42
Input	Modalities	DWI, ADC, FLAIR
Input	Patch size	3×128×128×128
Training	Batch size	1
Training	Maximum epochs	120
Training	Validation interval	5 epochs
Training	Early stopping patience	10 validation rounds
Optimizer	Optimizer	AdamW
Optimizer	Initial learning rate	1×10^{-4}
Optimizer	Main weight decay	1×10^{-5}
Optimizer	Gate parameter learning rate	0.1×main learning rate
Optimizer	Gate parameter weight decay	0
Scheduler	Scheduler	ReduceLROnPlateau
Scheduler	Factor / patience / min LR	$0.5 / 3 / 1 \times 10^{-6}$
Normalization	Normalization layer	Instance normalization
AMP	Mixed precision	Enabled
Gradient control	Gradient clipping	Max norm = 5.0
Loss	DiceCE loss	Enabled
Loss	UWB loss weight	0.4
Consistency	Base consistency weight	0.1
Consistency	Ramp-up epochs	20
Consistency	Uncertainty threshold	0.6
Gate	Gate temperature τ	1.2
Gate	Gate warm-up epochs	20
Gate regularization	Entropy regularization weight	1×10^{-3}
Gate regularization	Entropy decay epochs	60
MIGGAT	Attention heads	Single-head modality-interaction attention
MIGGAT	Modality nodes	DWI, ADC, FLAIR
MIGGAT	Projection ratio	0.5
MIGGAT	β	1.0
MIGGAT	Boundary enhancement coefficient	2.0

Category	Setting	Value
Data augmentation	Random flip	Probability 0.5
Data augmentation	Random 90° rotation	Probability 0.5, max k = 3
Data augmentation	Random affine	Probability 0.1
Data augmentation	Contrast adjustment	Probability 0.3, gamma 0.7–1.5
Inference	Sliding-window size	128 × 128 × 128
Inference	Sliding-window batch size	1
Inference	Sliding-window overlap	0.5
Ensemble	Ensemble strategy	Probability averaging of five fold-specific models
UWA	Top uncertainty ratio	Top 20%

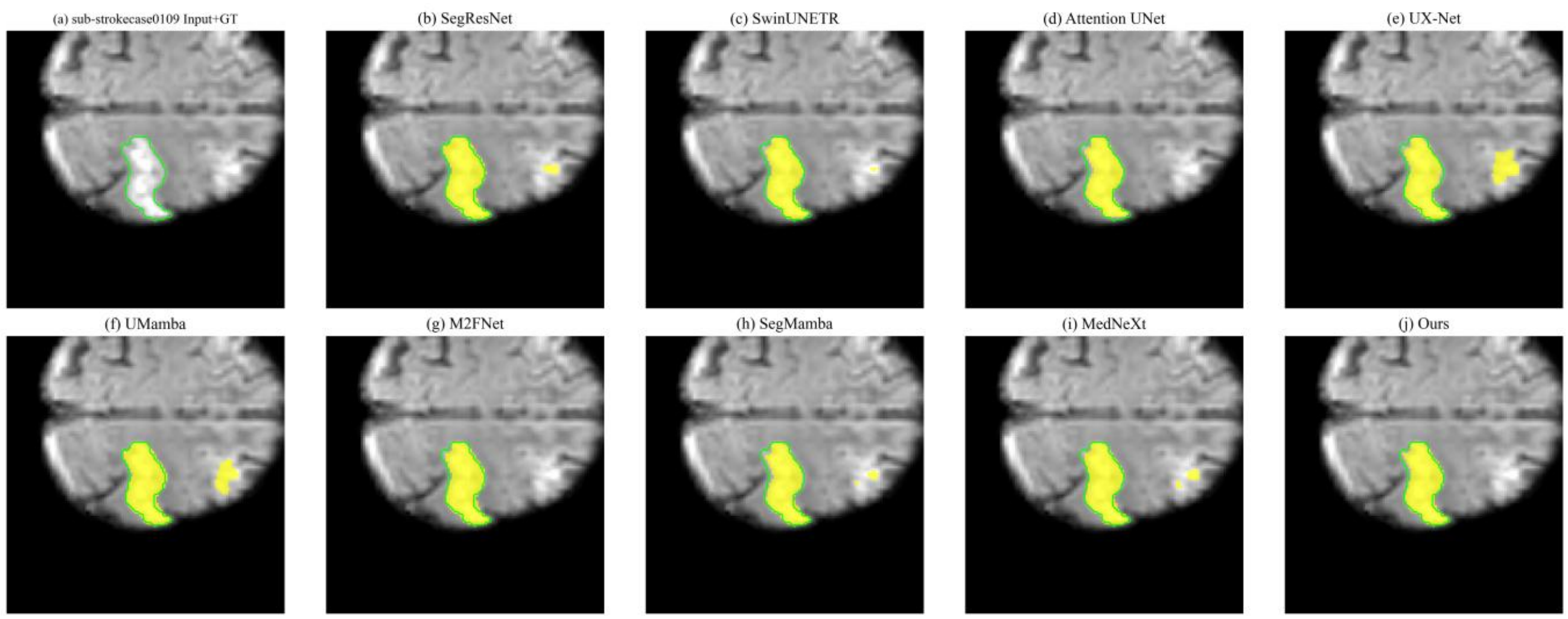

Supplementary Figure S1. Additional qualitative comparison of different models on a representative ISLES-2022 case (sub-strokecase0109). Panel (a) shows the input DWI slice and ground-truth contour, and panels (b)-(j) show the predictions of SegResNet, SwinUNETR, Attention UNet, UX-Net, UMamba, M2FNet, SegMamba, MedNeXt, and MBGS-UNet, respectively. In this case, most methods captured the main lesion extent, whereas MBGS-UNet showed cleaner suppression of scattered false-positive responses while preserving the primary lesion contour.

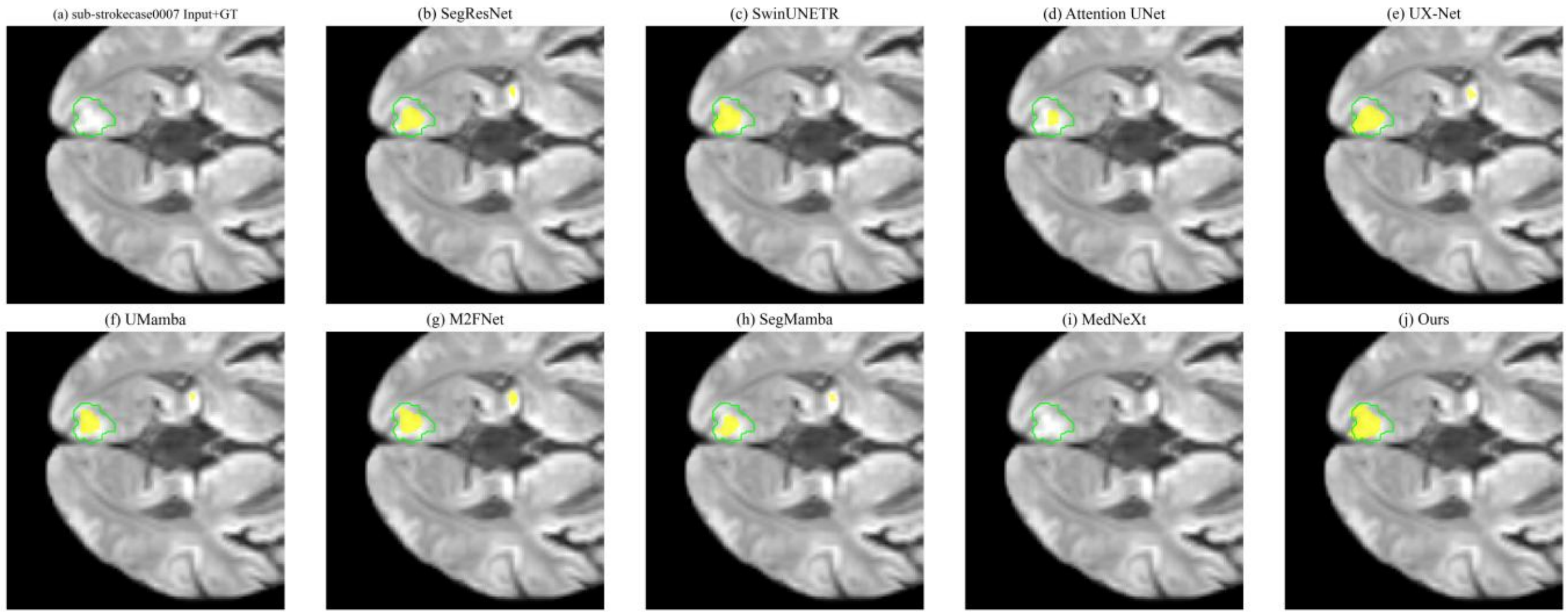

Supplementary Figure S2. Additional qualitative comparison of different models on a representative ISLES-2022 case (sub-strokecase0007). Panel (a) shows the input DWI slice and ground-truth contour, and panels (b)-(j) show the predictions of SegResNet, SwinUNETR, Attention UNet, UX-Net, UMamba, M2FNet, SegMamba, MedNeXt, and MBGS-UNet, respectively. In this case, several competing methods produced an additional scattered false-positive region, whereas MBGS-UNet preserved the main lesion extent while showing cleaner background suppression.

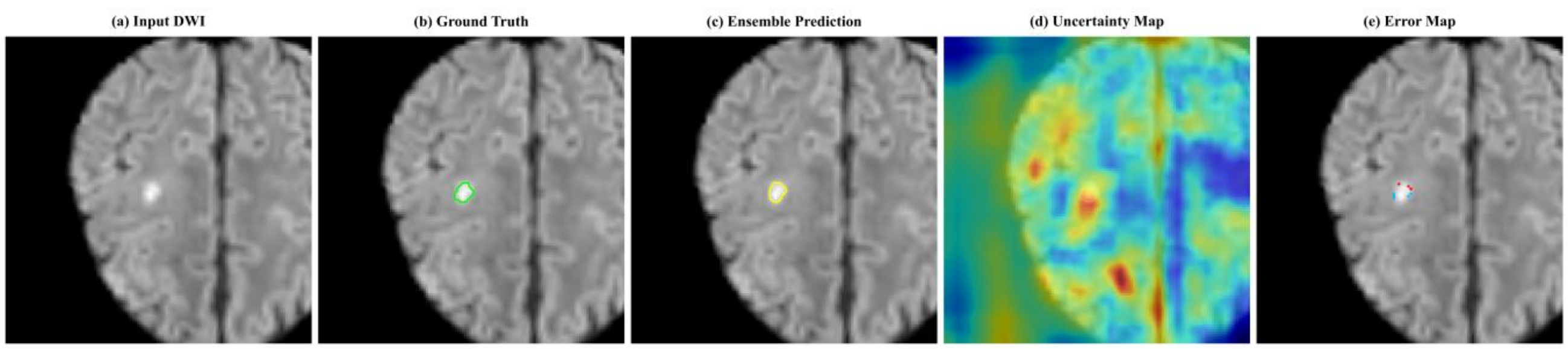

Supplementary Figure S3. Additional example showing the correspondence between uncertainty response and local prediction error. Panels (a)-(e) show the input DWI slice, ground truth, ensemble prediction, uncertainty map, and error map, respectively. In this case, the remaining prediction errors were mainly localized near the lesion region and showed substantial spatial overlap with the high-uncertainty response, supporting the potential value of uncertainty maps for qualitative error localization and result interpretation.

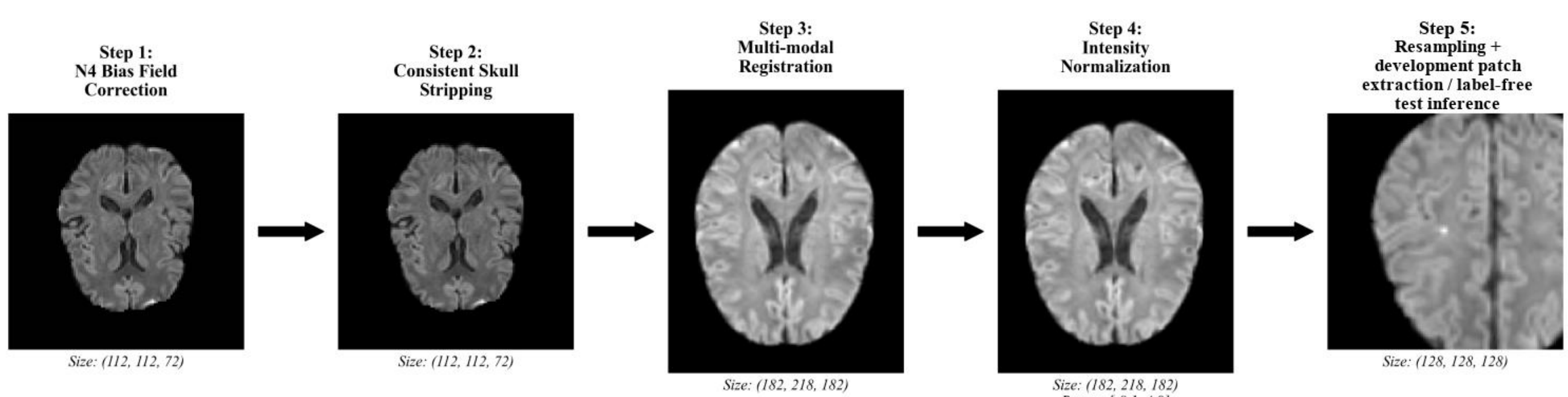

Supplementary Figure S4. Overview of the five-stage data preprocessing workflow. The workflow included bias correction, skull stripping, multimodal registration, intensity normalization, and 1 mm^3 resampling. After these common preprocessing steps, lesion-centered 128 × 128 × 128 patches were used for model development, whereas independent test-set inference was performed using label-free sliding-window inference with the same 128 × 128 × 128 window size.

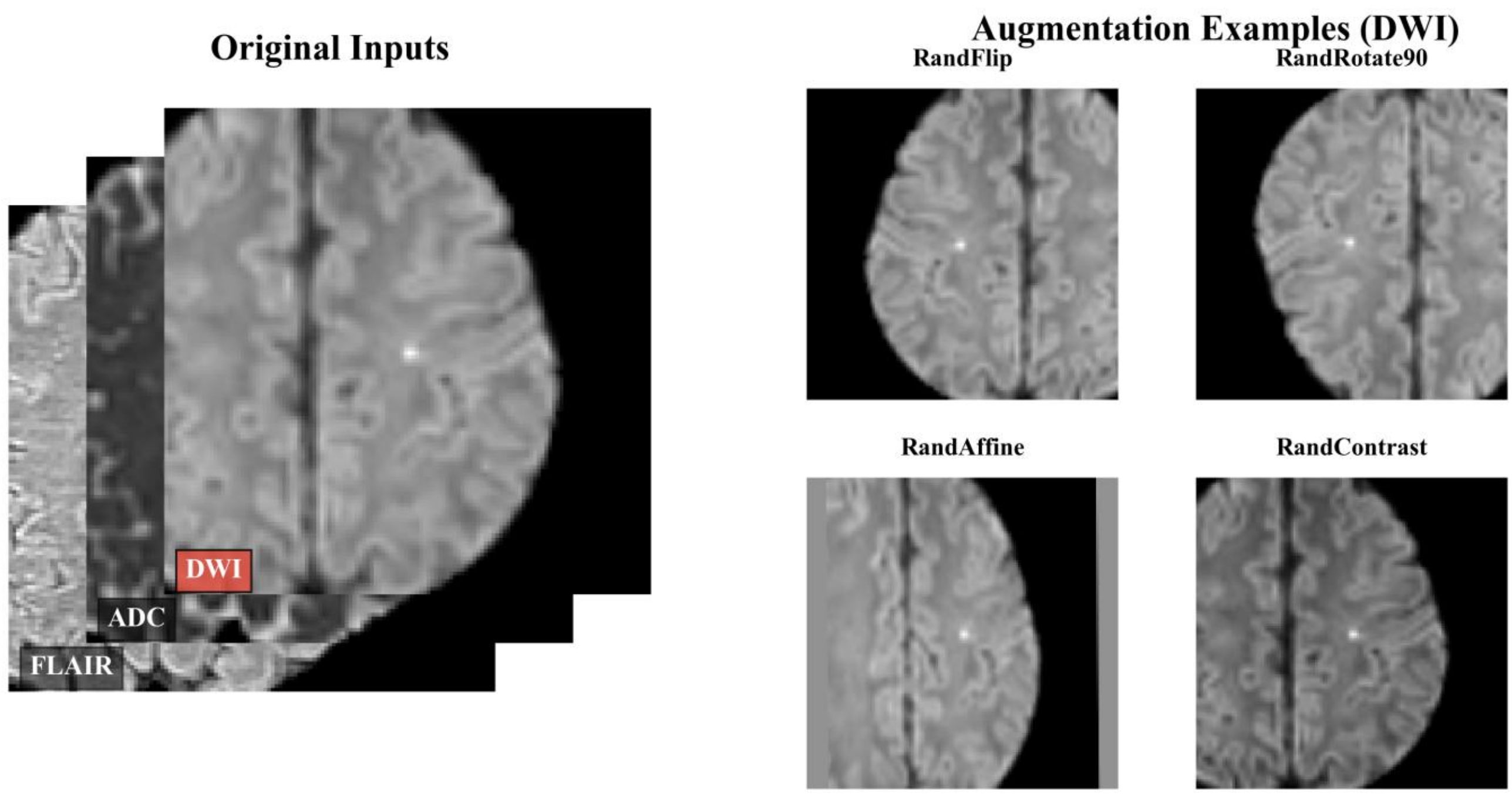

Supplementary Figure S5. Multimodal input and augmentation examples used for training. The left panel presents the multimodal input channels (DWI, ADC, and FLAIR). The right panel provides four examples of random augmentation applied to DWI, including flipping, rotation, affine transformation, and contrast adjustment.

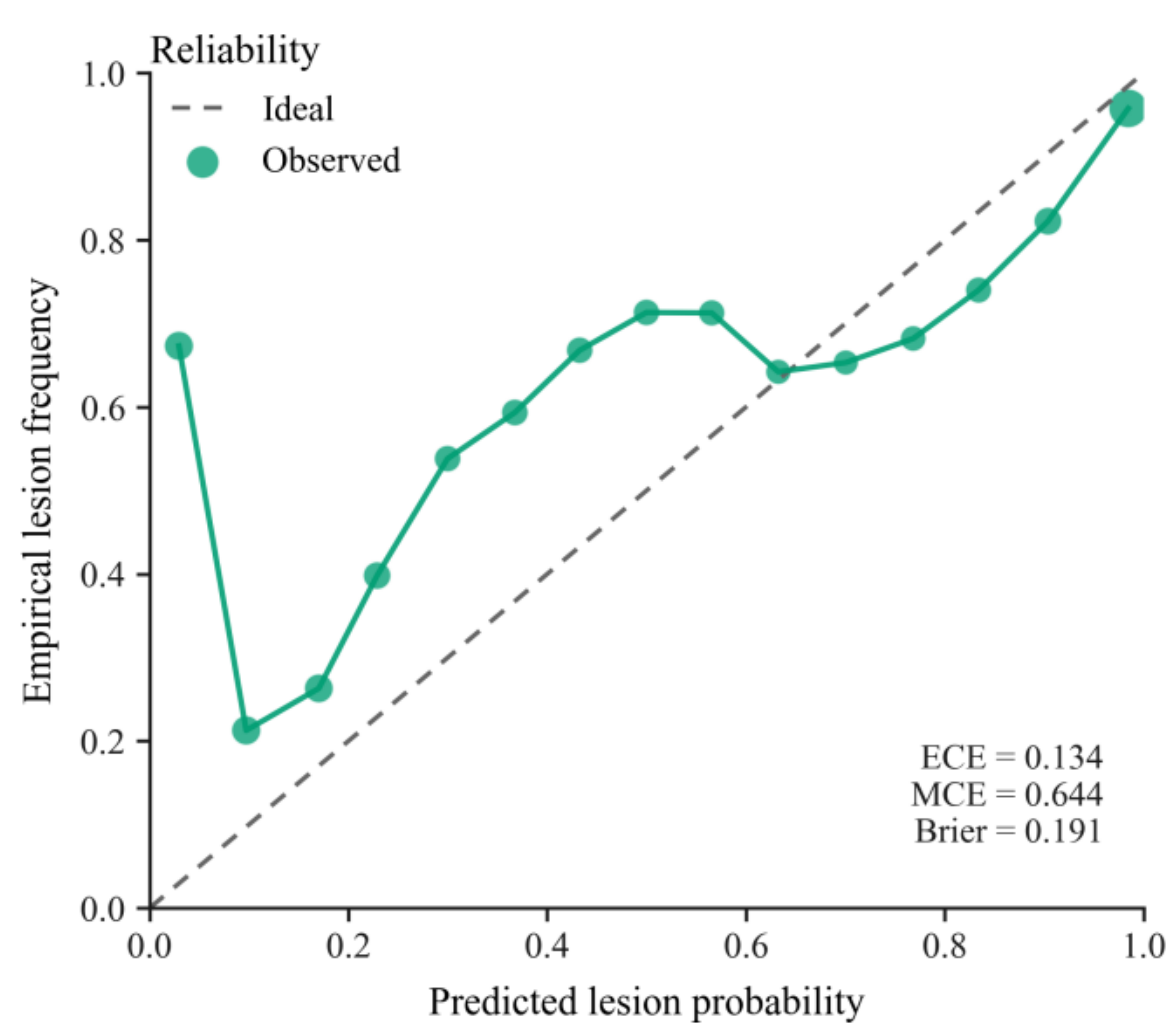

Supplementary Figure S6. Reliability analysis in the candidate lesion region. The diagram shows the relationship between mean predicted lesion probability and empirical lesion frequency across probability bins. The dashed diagonal line indicates perfect calibration, and the size of each point reflects the number of voxels in the corresponding bin. The ECE was 0.134, with MCE of 0.644 and Brier score of 0.191. This analysis was used as a supplementary calibration-related reference for characterizing the probability maps.

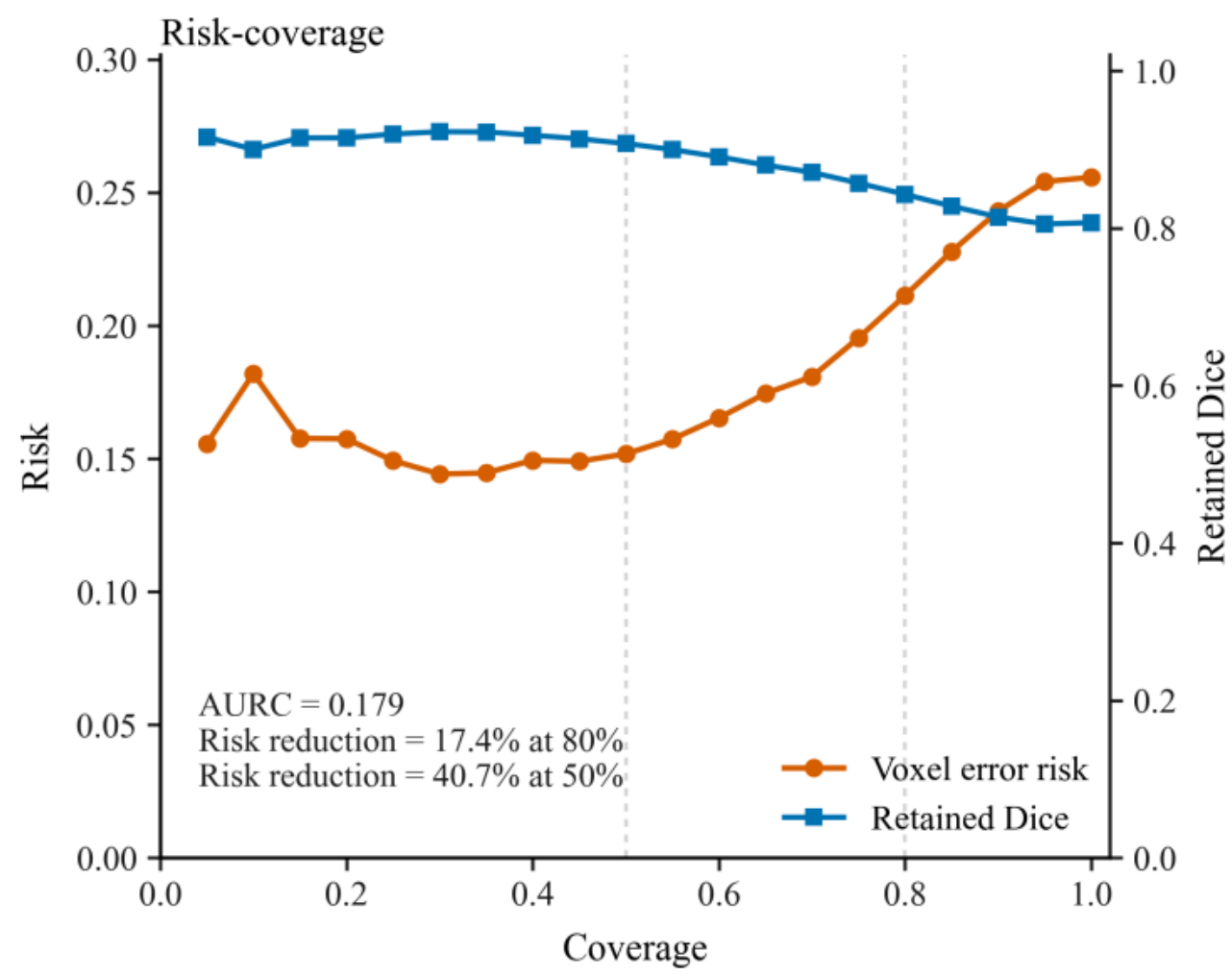

Supplementary Figure S7. Risk-coverage analysis based on predictive uncertainty. Voxels were ranked according to predictive uncertainty, and lower-uncertainty voxels were retained at different coverage levels. The voxel-wise error risk decreased when only lower-uncertainty voxels were retained. Compared with full coverage, the error risk was reduced by 17.4% at 80% coverage and by 40.7% at 50% coverage, suggesting that uncertainty was related to prediction reliability.

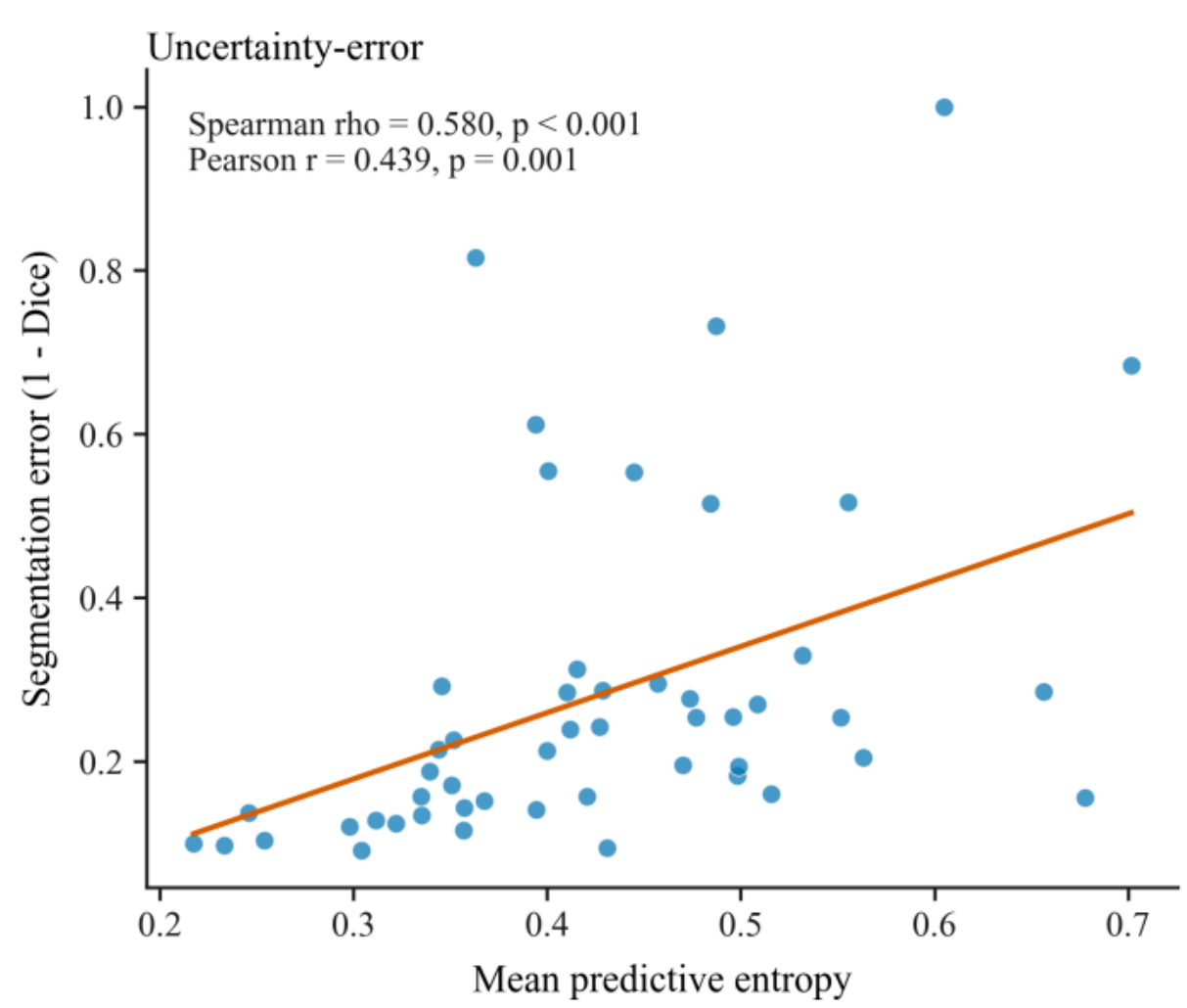

Supplementary Figure S8. Case-wise uncertainty-error correlation analysis. Each point represents one independent test case. The x-axis shows the mean predictive entropy in the candidate lesion region, and the y-axis shows segmentation error, defined as 1-Dice. A positive correlation was observed

between mean predictive entropy and segmentation error, with Spearman $\rho=0.580$ and $p=1.02\times10^{-5}$, indicating that cases with higher uncertainty tended to have less reliable segmentation results.
